# Homologous Recombination Deficiency in Ovarian and Breast Cancers: Biomarkers, Diagnosis, and Treatment

**DOI:** 10.3390/cimb47080638

**Published:** 2025-08-08

**Authors:** Bhaumik Shah, Muhammad Hussain, Anjali Seth

**Affiliations:** 1 Department of Pathology, Fox Chase Cancer Center, Temple Health, Philadelphia, PA 19121, USA; 2 Department of Pathology, Temple University Hospital, Temple Health, 3401 N Broad St., Philadelphia, PA 19140, USA

**Keywords:** homologous recombination deficiency (HRD), ovarian cancer, breast cancer, BRCA1/2, genomic scars, loss of heterozygosity (LOH), PARP inhibitors (PARPi), multi-omics, liquid biopsy, precision oncology

## Abstract

Homologous recombination deficiency (HRD) is a pivotal biomarker in precision oncology, driving therapeutic strategies for ovarian and breast cancers through impaired DNA double-strand break repair. This narrative review synthesizes recent advances (2021–2025) in HRD’s biological basis, prevalence, detection methods, and clinical implications, focusing on high-grade serous ovarian carcinoma (HGSOC; ~50% HRD prevalence) and triple-negative breast cancer (TNBC; 50–70% prevalence). HRD arises from genetic (*BRCA1/2*, *RAD51C/D*, *PALB2*) and epigenetic alterations (e.g., *BRCA1* methylation), leading to genomic instability detectable via scars (LOH, TAI, LST) and mutational signatures (e.g., COSMIC SBS3). Advanced detection integrates genomic assays (Myriad myChoice CDx, Caris HRD, FoundationOne CDx), functional assays (RAD51 foci), and epigenetic profiling, with tools like HRProfiler and GIScar achieving >90% sensitivity. HRD predicts robust responses to PARP inhibitors (PARPi) and platinum therapies, extending progression-free survival by 12–36 months in HGSOC. However, resistance mechanisms (*BRCA* reversion, *SETD1A/EME1*, *SOX5*) and assay variability (60–70% non-*BRCA* concordance) pose challenges. We propose a conceptual framework in Section 10, integrating multi-omics, methylation analysis, and biallelic reporting to enhance detection and therapeutic stratification. Regional variations (e.g., Asian cohorts) and disparities in access underscore the need for standardized, cost-effective diagnostics. Future priorities include validating novel biomarkers (SBS39, miR-622) and combination therapies (PARPi with ATR inhibitors) to overcome resistance and broaden HRD’s applicability across cancers.

## 1. Introduction

Homologous recombination deficiency (HRD) is a critical concept in cancer biology with significant therapeutic implications in precision oncology [1,2]. HRD refers to a state where cells cannot effectively repair double-strand DNA breaks using the homologous recombination repair (HRR) pathway, a precise mechanism that relies on a homologous DNA template to restore genomic integrity. HRD gained prominence with the discovery that mutations in the breast cancer genes *BRCA1* (Breast Cancer Gene 1) or *BRCA2* (Breast Cancer Gene 2) impair HRR [3,4]. These *BRCA1/BRCA2* mutations, linked to hereditary breast and ovarian cancers, lead to a distinct cancer phenotype. The complexity of HRD extends beyond just *BRCA*1/*BRCA*2 mutations, as it can arise from alterations in other HRR-related genes or epigenetic changes, leading to a broader HRD phenotype. For example, Ren et al. (2025) identified biallelic alterations in *BRCA1*/*BRCA2*, *RAD51C*, *RAD51D*, *PPP2R2A*, and *TP53* as key HRD drivers in Asian cohorts of ovarian and breast cancers, with chromosome-specific loss of heterozygosity (LOH) enhancing detection sensitivity [5]. Fiegl et al. (2024) highlighted *BRCA1* promoter methylation as a significant epigenetic contributor in high-grade serous ovarian carcinoma (HGSOC), predicting platinum sensitivity [6]. Zhao et al. (2023) and Wei et al. (2021) demonstrated that RNA methylation (e.g., N6-methyladenosine, m6A) regulates homologous recombination (HR) gene expression, contributing to HRD in ovarian cancer [7,8]. HRD undermines the functionality of both double-strand DNA break repair (DSBR) and synthesis-dependent strand annealing (SDSA), leading to profound genomic instability. This understanding is vital for developing targeted therapies that capitalize on the DNA repair deficiencies in HRD-positive cancers [1,2,3,4].

Historically, HRD has been linked to increased sensitivity to DNA-damaging agents like platinum-based chemotherapy and targeted therapies such as poly (ADP-ribose) polymerase inhibitors (PARPi), which exploit synthetic lethality by blocking base excision repair (BER) and overwhelming HR-deficient cells with unrepaired double-strand breaks (DSBs) [2,3]. Murai and Pommier (2023) emphasized PARPi’s efficacy through PARP1/2 trapping and catalytic inhibition, with HRD signatures identifying a broader range of treatment candidates [9]. The clinical relevance of HRD emerged prominently in cancers such as ovarian, breast, pancreatic, and prostate cancer, where identifying HRD-positive patients can guide treatment decisions [1]. Abbasi et al. (2025) developed HRProfiler, a WES-based machine learning tool for improved HRD detection in breast and ovarian cancers, independently evaluated by Lim and Ju (2025) for clinical utility, while Leman et al. (2025) suggested serum miR-622 could serve as a minimally invasive predictive independent biomarker for PARPi response in newly diagnosed and recurrent HGSOC [10,11,12]. However, ongoing challenges persist in defining HRD consistently, as its scope has expanded beyond *BRCA*1/2 mutations to include a wider array of genetic, epigenetic and functional defects, necessitating robust diagnostic tools and standardized criteria.

The review provides a comprehensive overview of HRD, bridging its biological basis, prevalence, and clinical relevance in ovarian and breast cancers. It offers updates and highlights from recent studies, examining a spectrum of HRD analysis and estimation methods—including genomic assays, functional tests, and emerging epigenetic profiling—while addressing diagnostic challenges and emphasizing the need for further research to refine HRD assessment and its clinical application. It also proposes a refined conceptual model (Section 10, integrating genomic assays (e.g., Myriad myChoice CDx, FoundationOne CDx, HRDetect), functional assays (e.g., RAD51 foci), and epigenetic analysis (e.g., *BRCA1*/*RAD51C* methylation) to capture the full HRD spectrum, including non-*BRCA* defects. Notably, Sahajpal et al. (2023) and Magadeeva et al. (2023) introduced high-sensitivity tools like optical genome mapping (OGM) and high-resolution array-based comparative genomic hybridization (aCGH), respectively, while Marconato et al. (2025) reviewed advanced testing platforms expanding PARPi eligibility [13,14,15]. The Archer™ VARIANTPlex and Affymetrix OncoScan assays offer scalable solutions, potentially integrating DNA and RNA methylation profiling to enhance HRD detection [16,17]. Furthermore, Sweatman et al. (2025) and Ghosh et al. (2025) highlighted resistance mechanisms involving *SETD1A*-dependent *EME1* transcription and *SOX5*, underscoring the need for resistance-informed assays [18,19]. Section 2 and Section 3 elucidate HRD’s molecular mechanisms, Section 4 defines biomarkers, Section 5, Section 6 and Section 7 quantify prevalence, Section 8 evaluates detection methods, Section 9 addresses clinical challenges, Section 10 and Section 11 discuss therapeutic implications, and Section 12, Section 13 and Section 14 outline future directions, emphasizing standardization equitable access and emerging diagnostic innovations.

### Methodology

This narrative review was compiled through a comprehensive analysis of the peer-reviewed literature from PubMed, using search terms such as “homologous recombination deficiency,” “ovarian cancer,” “breast cancer,” “(PARPi),” “genomic instability,” and “epigenetic profiling.” Studies from 2022 to 2025 were prioritized to reflect recent advances in HRD biology, detection, and therapeutics, focusing on HGSOC and triple-negative breast cancer (TNBC). Selection criteria emphasized relevance to HRD biomarkers, diagnostic methodologies, and clinical applications. Data were synthesized to provide a cohesive overview of HRD’s molecular basis, prevalence, detection methods, therapeutic strategies, and challenges. Study quality was informally assessed based on cohort size, methodological rigor, and journal reputation. Limitations of the narrative approach, such as the lack of systematic quality assessment (e.g., PRISMA), are acknowledged for transparency.

## 2. HRD Pathway

HR is a critical, high-fidelity DNA repair pathway that uses a homologous DNA sequence (typically from a sister chromatid) as a template to repair DSBs to maintain genomic stability. This process is most active during the S and G2 phases of the cell cycle, when sister chromatids are readily available [20]. It initiates with the precise recognition of the lesion by sensor proteins, notably the MRN complex (*MRE11-RAD50-NBS1*) [20]. Following this detection, end resection, mediated by proteins such as *CtIP* and *EXO1*, generates 3′ single-stranded DNA (ssDNA) tails, which are essential for strand invasion [21]. Subsequently, RAD51, a key recombinase, coats these ssDNA tails, facilitating strand invasion, where one tail pairs with the homologous DNA duplex, typically the sister chromatid, forming a displacement loop (D-loop) [22]. The D-loop, created by single-end invasion (SEI), may enter the DSBR pathway and form a double Holliday junction, where the invading strand and the homologous DNA are crossed over [22,23]. This invasion allows DNA polymerase to utilize the undamaged strand as a template to accurately repair the break. The process culminates in Holliday junction resolution, which can result in either crossover (CO) or non-crossover (NCO) products, ultimately completing the repair [23]. This intricate and highly regulated pathway ensures genomic stability by faithfully restoring DNA integrity, thereby minimizing the accumulation of deleterious mutations.

HR is not a singular, uniform process; it encompasses variations, most notably the DSBR pathway and SDSA, which lead to distinct outcomes [24,25]. The DSBR pathway involves the formation of double Holliday junctions, which are crucial intermediate structures formed when two homologous DNA duplexes are linked by crossed strands during the repair process [22,23]. Resolution of these Holliday junctions involves the cleavage of these cross-stranded links, carried out by Holliday junction resolvases. The manner in which resolvases cut the DNA strands determines whether the outcome of resolution is CO or NCO products [23,25]. Resolution to CO requires symmetric cleavage of both Holliday junctions in opposite orientations by a resolvase, while resolution to NCOs can also be achieved by the resolvase through cleavage of both junctions in the same orientation. CO events result in the exchange of genetic material between homologous chromosomes, as segments of DNA from one molecule are swapped with corresponding segments from the other. In SDSA, however, the process involves strand invasion and DNA synthesis, but the newly synthesized strands are displaced and anneal to the other broken end, without forming Holliday junctions [25,26]. This pathway primarily results in NCO products. DSBR, a robust repair mechanism, can contribute to genetic diversity through CO events, which are particularly important during meiosis. However, the potential for crossovers means it can also lead to LOH. SDSA is a preferred pathway for maintaining genetic stability, as it avoids CO events [25,26,27]. It is crucial for accurate DNA repair in somatic cells, where minimizing genetic rearrangements is essential, and the parental arrangement of genes is preserved. The choice between DSBR and SDSA depends on the cellular context, such as the cell cycle phase and the type of cell (somatic vs. germline). During meiosis, DSBR is crucial for generating genetic diversity in gametes [28]. In somatic cells, SDSA is often favored to preserve genomic integrity. Thus, SDSA promotes genomic stability, while DSBR has the potential to alter the genetic makeup of the cell. Table 1 summarizes key differences between DSBR and SDSA, highlighting their distinct roles in genomic stability and their relevance to HRD, with implications for assay design targeting these pathways.

HRD represents a cellular phenotype characterized by the compromised function of the HR DNA repair pathway, resulting in the inefficient repair of DSBs and subsequent genomic instability. When this pathway is deficient—due to mutations, deletions, or the silencing of its associated genes—cells resort to error-prone repair mechanisms, such as non-homologous end joining (NHEJ), leading to accumulated DNA damage and genomic instability. Both normal and cancerous cells possess intricate DDR pathways (see Table 2), which are essential for preserving genomic integrity by repairing diverse DNA lesions. These pathways are critical for cellular survival and proper function. Murai and Pommier (2023) emphasized HRD’s role in synthetic lethality with PARPi, driven by PARP1/2 trapping and catalytic inhibition, with reliance on error-prone NHEJ pathways exacerbating genomic instability [9]. Wu et al. (2024) showed that m6A and m5C RNA methylation regulate R-loop formation, impacting HR efficiency and contributing to HRD phenotypes in breast and ovarian cancers [29]. HRD shifts repair to error-prone pathways, driving tumorigenesis, which can be detected through molecular assays, functional tests, and epigenetic analyses, such as promoter methylation. The Archer™ VARIANTPlex HRD workflow uses allele-specific copy number (ASCN) entropy and NHEJ metrics to quantify genomic instability, potentially enhanced by integrating DNA and RNA methylation profiling to capture epigenetic contributions to HRD [16].

HRD often arises from genetic or epigenetic alterations in key HRR genes, most notably *BRCA1* and *BRCA2*, though it extends to other genes, such as *ATM*, *ATR*, *RAD51*, *RAD50*, *PALB2*, *BARD1*, *BRIP1*, *CDK12*, *CHEK1*, *CHEK2*, *EMSY*, *FANCA*, *FANCL*, *H2AX*, *MRE11*, *NBN*, *RPA*, *RAD51B*, *RAD51C*, *RAD51D*, and *RAD54L* [2,12,22,25,28,34]. The condition is not solely defined by these mutations; rather, it encompasses a broader phenotype in which the HRR pathway is functionally impaired, leading to genomic instability. This disruption affects both the DSBR and SDSA subpathways, as each relies on functional HRR machinery [9,35,36,37]. The DSBR pathway, involving Holliday junction formation and resolution, is particularly vulnerable in HRD [25]. Deficiencies in HRR genes associated with the DSBR pathway impair the precise processing and resolution of Holliday junctions, leading to increased genomic instability, higher rates of chromosomal abnormalities, and a potential for deleterious crossover events [9,22,25,36,37]. Although the SDSA pathway avoids Holliday junction formation, it still requires the core HR machinery and key HRR proteins for strand invasion and DNA synthesis. HRD compromises the efficiency of SDSA, resulting in increased reliance on alternative, error-prone repair pathways [9,35].

## 3. Genes and Mechanisms Involved in Homologous Recombination Deficiency in Ovarian and Breast Cancers

HRD is defined by impaired double-strand break (DSB) repair, resulting in genomic instability. This deficiency often arises from genetic or epigenetic alterations in key HRR genes, most notably *BRCA1* and *BRCA2*, though it extends to other genes, such as *ATM*, *RAD51*, *PALB2*, *BARD1*, *BRIP1*, *CDK12*, *CHEK1*, *CHEK2*, *FANCL*, *RAD51B*, *RAD51C*, *RAD51D*, and *RAD54L* [34,38,39]. In the presence of DSBs, BRCA1 and BRCA2 collaborate with other HR proteins to facilitate repair. BRCA1 facilitates DNA end resection and recruits repair factors, while BRCA2 loads RAD51 onto single-stranded DNA, forming nucleoprotein filaments that drive homologous strand invasion [2,4]. PALB2 acts as a bridging protein, connecting *BRCA1* and *BRCA2* [30]. ATM, a key sensor of DSBs, initiates a signaling cascade by phosphorylating downstream targets, including CHEK2, which activates cell cycle checkpoints and promotes DNA repair [38]. The MRN complex (MRE11, RAD50, NBN) stabilizes broken DNA ends and initiates the repair process [25]. Fanconi anemia (FA) proteins, such as FANCD2 and FANCI, contribute to DNA repair, particularly for ICLs, in coordination with the HR pathway [30]. Ren et al. (2025) identified biallelic alterations in *BRCA1*, *BRCA2*, *RAD51C*, *RAD51D*, *PPP2R2A*, and *TP53* as key HRD drivers in Asian cohorts of ovarian and breast cancers, enhancing therapeutic response prediction [5]. Fiegl et al. (2024) highlighted *BRCA1* promoter methylation as a significant epigenetic mechanism inducing HRD in HGSOC, mimicking genetic defects [6]. Zhao et al. (2023) and Wei et al. (2021) identified m6A RNA methylation regulators (e.g., *METTL3*, *ALKBH5*, *IGF2BP2*) as novel contributors to HRD by modulating HR gene expression in ovarian cancer [7,8]. Pae et al. (2024) demonstrated that PLK1 overexpression suppresses HR via kinase-dependent inhibition of RAD51 foci formation, enhancing HRD in breast and ovarian cancers [40]. Wu et al. (2024) showed that m6A and m5C RNA methylation regulate R-loop formation, impairing HR efficiency and contributing to HRD phenotypes [29]. HRD leads to chromosomal and sub-chromosomal aberrations, including structural abnormalities, copy number variations, and insertions/deletions (indels), elevating the risk of malignancy [41,42].

A hallmark of HRD is genomic instability, manifesting as “genomic scars” (see Section 4 for definitions), which reflect reliance on error-prone repair mechanisms like NHEJ or single-strand annealing (SSA) [9,25,34,35]. Biallelic inactivation of HRR genes, particularly *BRCA1*/*BRCA2*, is a key driver of HRD, and reporting this status enhances prediction of therapeutic response to PARPi [5,9]. Murai and Pommier (2023) emphasized HRD’s role in synthetic lethality with PARPi, driven by reliance on error-prone NHEJ pathways [9]. DNA damage response (DDR) pathways, summarized in Table 2, are essential for genomic integrity. Key DDR mechanisms include HRR, BER, nucleotide excision repair (NER) with subpathways global genome NER (GG-NER) and transcription-coupled NER (TC-NER), mismatch repair (MMR), NHEJ, translesion synthesis (TLS), and interstrand crosslink (ICL) repair. DDR integrates with cell cycle checkpoints, with proteins like ATM and ATR signaling damage to halt progression. High-fidelity pathways (HRR, BER, NER, MMR, ICL repair) minimize errors, while NHEJ and TLS are error prone. HRR and NHEJ are phase-specific (S/G2 and G1, respectively), while BER and NER operate throughout the cycle [22,23,24,25,29,30,31,32]. The Archer™ VARIANTPlex HRD workflow uses ASCN entropy and NHEJ metrics to quantify genomic instability, potentially enhanced by integrating DNA and RNA methylation profiling to capture epigenetic contributions to HRD [16].

## 4. Biomarkers of HRD

Accurate identification of HRD is critical for diagnosis and treatment stratification in ovarian and breast cancers. The landscape of HRD biomarkers has expanded considerably, now encompassing genetic, genomic, and epigenetic alterations that reflect the diverse mechanisms underlying HRR dysfunction. Germline and somatic mutations in *BRCA1* and *BRCA2* remain the primary and most recognized drivers of HRD. However, tumors lacking *BRCA1/BRCA2* mutations can display “BRCAness,” mimicking HRD-like features through alterations in other HRR genes such as *RAD51C*, *RAD51D*, *BRIP1*, and *PALB2* [4]. A hallmark of HRD is the accumulation of characteristic genomic scars, including LOH, telomeric allelic imbalance (TAI), and large-scale state transitions (LST), which collectively reveal DNA damage and repair defects caused by HRD [4,5,9,11,12,25,41,42,43]. These genomic features are routinely quantified by clinical assays and are central to HRD scoring systems that guide therapeutic decisions. Whole-genome sequencing (WGS) enhances the identification of distinct mutational signatures, such as COSMIC Signature 3, improving the precision of HRD detection [44,45].

Beyond DNA sequence alterations, epigenetic mechanisms play a significant role in HRD. Promoter hypermethylation of key HRR genes, especially *BRCA1* and *RAD51C*, can silence gene expression and induce HRD, with prevalence reaching up to 60% in TNBC and 1–15% in ovarian cancer [46,47]. Fink et al. (2025) validated a quantitative next-generation sequencing (NGS) assay for measuring *BRCA1* and *RAD51C* promoter methylation in breast and ovarian cancers [47]. This underscores the importance of targeted methylation analysis in mutation-negative cases to identify additional HRD-positive patients. Recent studies have identified RNA methylation regulators, such as m6A and m5C modifications (e.g., *METTL3*, *ALKBH5*, *IGF2BP2*), as novel epigenetic biomarkers linked to HRD-related pathways and prognosis, with these modifications shown to regulate R-loop formation and impact homologous recombination efficiency [7,8,29]. Ren et al. (2025) reported biallelic alterations in *BRCA1*, *BRCA2*, *RAD51C*, *RAD51D*, *PPP2R2A*, and *TP53* as key HRD drivers in Asian cohorts, with chromosome-specific LOH (e.g., 8p, 17p) enhancing detection sensitivity [5]. Fiegl et al. (2024) demonstrated *BRCA1* methylation in 11% of HGSOC, exclusively in *BRCA1*-wild-type cases, with HRD scores comparable to *BRCA1*-mutated tumors and predictive of platinum sensitivity [6].

The biomarker landscape continues to evolve with minimally invasive approaches, such as serum miR-622 and metabolomic profiling, which have shown promise in predicting platinum and PARPi response, particularly in HGSOC [12]. Technological advances have further expanded the toolkit for HRD detection. HRProfiler, a whole-exome sequencing (WES)-based tool developed by the Alexandrov Lab (University of California San Diego, La Jolla, CA, USA), using six mutational features (e.g., LOH: 1–40 Mb, DEL.5.MH), achieves AUC > 0.90 for HRD detection in breast and ovarian cancers [10,11], while DirectHRD, developed by the Broad Institute and Dana-Farber Cancer Institute (Cambridge and Boston, MA, USA), leverages microhomology deletions for 100% sensitivity in tumor biopsies and 90% in low tumor fraction cfDNA [48]. Low-pass WGS assays (GSscan, shallowHRD, AcornHRD) have demonstrated high accuracy (AUC 0.980–0.997) for HRD detection in breast cancer [49,50]. Oncomine has shown 95.8% accuracy in predicting HRD in HGSOC [51]. OGM detects 70.8% additional HRD signatures (e.g., translocations) compared to chromosomal microarray analysis (CMA) and NGS [13], and high-resolution aCGH offers 77% sensitivity for HRD in ovarian cancer, with mutation frequency correlating with HRD scores [14]. HRDsig positivity has been reported in 82% of germline or somatic g/s*BRCA1/BRCA2* or germline *PALB2*-mutated breast cancers and 16.5% of HRR wild-type cancers [52]. Batalini et al. (2023) found that approximately 23% of metastatic breast cancers were HRDsig-positive [53]. Additional biomarkers, such as PLK1 overexpression, *SETD1A/EME1*, and *SOX5*, have been implicated in modulating HRD and resistance to PARPi [18,19,39]. The Archer™ VARIANTPlex HRD workflow uses ASCN entropy and NHEJ metrics, potentially enhanced by integrating DNA and RNA methylation, while OncoScan’s high-resolution SNP arrays (220,000 SNPs) offer robust CNA/LOH detection for HRD [16,17].

Collectively, these advances have transformed HRD detection into a comprehensive, multi-layered approach integrating genetic, genomic, epigenetic, and functional data. Table 3 summarizes the key biomarkers and genomic signatures of the HRD phenotype, providing definitions, characteristics, and clinically relevant thresholds (e.g., gLOH ≥ 14%, NtAI ≥ 22, LST ≥ 15), serving as a concise reference for research and clinical practice [40,41,42,54,55,56]. This integrated framework enhances diagnostic accuracy and broadens the population of patients who may benefit from targeted therapies, marking a significant advance in precision oncology.

Key Points: Section 4—Biomarkers of HRD

- *BRCA1*/*BRCA2* mutations and genomic scars (LOH, TAI, LST) are primary HRD biomarkers.
- Non-*BRCA* HRR genes (*RAD51C*, *PALB2*) and *BRCA1* methylation expand HRD detection.
- m6A/m5C RNA methylation (*METTL3*, *ALKBH5*) modulates HRD via R-loop accumulation.
- Serum miR-622 and HRProfiler enhance non-invasive and precise HRD identification.

## 5. Prevalence of HRD in Ovarian and Breast (TNBC) Cancers

HRD is a critical factor in ovarian and breast cancers, with prevalence varying by tumor subtype and genetic background. The Cancer Genome Atlas (TCGA) data indicate that ~50% of HGSOC cases exhibit genetic and epigenetic alterations in HR pathway genes, notably *BRCA1* and *BRCA2* [29]. In TNBC, HRD prevalence ranges from 50% to 70%, driven by *BRCA1/BRCA2* mutations and genomic instability [50]. HGSOC’s high HRD rate reflects genomic instability, while TNBC’s is linked to aggressive behavior and BRCA defects. Biallelic inactivation and promoter methylation significantly contribute, as detailed in Section 4, Section 6 and Section 7. Table 4 summarizes HRD prevalence in HGSOC and breast cancer subtypes, with genetic, epigenetic drivers and regional variations detailed in Table 4, Table 5 and Table 6.

**Table 4:** HRD prevalence in HGSOC and breast cancer subtypes.

Cancer Type/Subtype	HRD Prevalence	Key Studies	Drivers/Additional Factors
Ovarian (HGSOC)			
HGSOC	~50%	TCGA (2011) [57]	19% germline *BRCA1*/*BRCA2*, 4–5% somatic, 15% epigenetic silencing
HGSOC	50–55%	Andrikopoulou et al. (2022) [58]	Germline: 13–15%, Somatic: 22% (*BRCA1*), 2% (*BRCA2*), NGS-based
HGSOC	50–51%	Quesada et al. (2022, 2025) [59,60]	Germline + Somatic: 20–25%, epigenetic silencing
Chinese HGSOC	50–52%	Min Wang et al. (2023) [61]	*BRCA1*/*BRCA2*: 20%, CNVs: 15–20%, platinum response
HGSOC	48–53%	Barnicle et al. (2024) [62]	Genomic instability (LOH, LST)
HGSOC	45–55%	Andrews et al. (2024) [63]	Variability in non-*BRCA* HRD detection
HGSOC	49–52%	Weichert et al. (2022) [64]	Optimized NGS concordance
HGSOC	51%	Wu et al. (2020) [65]	TAI, LOH, LST in combined HRD score
HGSOC	~47.5–50%	Fumagalli et al. (2022) [66]	In-house AmoyDx vs. Myriad concordance
HGSOC	55–60%	Capoluongo et al. (2022) [67]	Genomic + functional assays
HGSOC	49–53%	Christinat et al. (2023) [68]	Normalized LST, olaparib response
HGSOC	~50%	Fiegl et al. (2024) [6]	11% *BRCA1* methylation, 83% HRD-positive in methylated tumors
HGSOC	33.2% HRR alterations	Ren et al. (2025) [5]	92.2% biallelic *BRCA1*/*BRCA2*, chromosome 8 LOH, LST, TAI
HGSOC	64.40%	Kang et al. (2024) [51]	32.2% *BRCA1*/*BRCA2* pathogenic variants, Oncomine GIM, 95.8% accuracy
HGSOC	63.3% (19/30)	Magadeeva et al. (2023) [14]	36.7% *BRCA1*/*BRCA2* mutations, *TP53* mutations
**Breast Cancer**			
TNBC	50–70%	Lenz et al. (2023) [69], Jacobson et al. (2023) [70], Zhang et al. (2022) [71], Lim et al. (2023) [72], Xiao Liu et al. (2022) [73], Pan et al. (2024) [74], Jeon et al. (2025) [52]	*BRCA1*/*BRCA2* mutations, replication stress, mutational signatures
HER2-positive	5–40%	Yndestad et al. (2023) [75], Lenz et al. (2023) [69], Jeon et al. (2025) [52]	*BRCA1*/*BRCA2*, genomic instability; 5% of non-TNBC if HRD strictly defined (mutational + methylation) [75]
Luminal A	5–25%	Lenz et al. (2023) [69], Engebrethsen et al. (2023) [76]	Diverse HR gene defects
Luminal B	20–35%	Lenz et al. (2023) [69], Jacobson et al. (2023) [70]	*BRCA1*/*BRCA2*, other HR genes
HR+/HER2-	15–20%	Yndestad et al. (2023) [75], Ballot et al. (2022) [77], Jeon et al. (2025) [52]	Non-*BRCA* HR alterations
Male Breast Cancer	~30%	André et al. (2020) [78]	*BRCA2*/*RAD51C* hypermethylation

Notes: Combines prevalence data for HGSOC and breast cancer subtypes, referencing Section 4 for biomarkers. Larger cohorts (e.g., Barnicle et al. [62]) enhance reliability; smaller studies (e.g., Andrikopoulou et al. [58]) may introduce bias. Prevalence aligns at ~50% for HGSOC and 50–70% for TNBC, with non-*BRCA* mechanisms (e.g., methylation, CNVs) filling the gap [5,6]. Multi-scale approaches improve detection sensitivity [51].

## 6. HRD in Serous Ovarian Cancer (HGSOC)

TCGA data show germline *BRCA1/BRCA2* mutations in ~19% of HGSOC patients, with somatic mutations adding 4–5% and epigenetic silencing (e.g., *BRCA1* promoter hypermethylation) affecting up to 15% [57]. Biallelic inactivation occurs in over 80% of *BRCA*-associated HGSOC, enhancing PARPi response prediction. HRD prevalence in HGSOC is consistently estimated at 48–55%, supported by genomic instability patterns (LOH, LST, TAI), structural variations (SVs), copy number alterations (CNAs), and functional assays [5,6,14,61,62,63,64,65,66,67,68].

Ren et al. (2025) reported 33.2% HRR gene alterations, with 92.2% of *BRCA1/BRCA2* alterations biallelic and chromosome-specific LOH (e.g., 8p, 17p) prevalent in Asian cohorts [5]. Fiegl et al. (2024) noted 11% *BRCA1* methylation, with 83% of methylated cases HRD-positive, predicting 99% platinum sensitivity [6]. Kang et al. (2024) confirmed 64.4% HRD positivity using Oncomine, with 32.2% harboring *BRCA1/BRCA2* pathogenic variants [51]. Magadeeva et al. (2023) reported 63.3% HRD prevalence, with 36.7% *BRCA1/BRCA2* mutations and *TP53* mutations in all HRD-positive cases [14]. Variability in non-*BRCA* HRD detection and assay methodologies can lead to underestimation, with inconclusive results affecting 5–10% of cases [63,79,80]. Standardized assays, including methylation and biallelic status assessment, are critical for accurate patient stratification, as discussed in Section 9.

## 7. HRD in Breast Cancer (TNBC and Other Subtypes)

HRD prevalence varies across breast cancer subtypes, with TNBC exhibiting the highest rates (50–70%), followed by HER2-positive (30–40%), luminal A (15–25%), luminal B (20–35%), and HR+/HER2− (15–20%) [75]. In male breast cancer, HRD prevalence is ~30%, driven by epigenetic silencing of *BRCA2* and *RAD51C* [78]. In a study of 3388 patients, 48.5% of pathogenic variants were in *BRCA1* (24%) and *BRCA2* (24.5%), with *CHEK2* (11.7%), *ATM* (9.7%), and *PALB2* (9.3%) also contributing. Among the 253 cases of TNBC in this cohort, somatic *BRCA1/BRCA2* mutations were present in ~3.5% of sporadic TNBC [81]. Fujisawa et al. (2021) reported a 20% HRD prevalence of g*BRCA1/BRCA2* mutations in a Japanese TNBC cohort, while Koh et al. (2022) found gBRCA1/2 in 10.6% overall and 11.6% in the TNBC subgroup of an Asian cohort (including Japanese) [82,83]. Notably, when larger multi-gene panels are used- capturing rare variants in *BRIP1* and *BARD1*-the prevalence in TNBC increases to (50–60%). Torres-Esquius et al. (2024) studied patients with germline *RAD51C/D* mutations and found that in untreated *RAD51D*-associated breast cancers within their cohort, 66.7% exhibited functional HRD and 90.0% exhibited genomic HRD, respectively. [84]. Using a strict definition (HRD-S), Engebrethsen et al. (2023) and Yndestad et al. (2023) found HRD in 47% of TNBC and 5% of non-TNBC tumors; with a broader definition (HRD-W), the rates increased to 59% in TNBC and 23% in non-TNBC [75,76]. In the study, HRD-S was defined by a high HRDetect score (≥0.7) or functional assay confirmation, encompassing *BRCA1/2* mutations, promoter methylation, and clear HRD features. Conversely, HRD-W included *BRCA1/2* mutations alongside HRDetect using a lower threshold or incorporated other repair genes and additional genomic or clinical factors. Feng et al. (2023) reported 35% HRD prevalence using genomic scar scores (GSS) in a cohort of 147 chinese breast cancer patients, with a significantly higher rate of HRD positivity was observed in TNBC (60.5%) compared to other subtypes like Luminal A (5.3%), Luminal B (HER2-) (28.8%), and Luminal B (HER2+) (31.6%) [81]. Jacobson et al. (2023) identified HRDsig in 16.5% of HRR wild-type cancers, with 30% in TNBC, 17% in ER+/HER2-, and 8.7% in HER2+ [70]. Lim et al. (2023) reported 50% HRD prevalence using machine learning-based mutational signature analysis, with TNBC showing elevated rates [72]. Batalini et al. (2023) observed 5% HRD in non-TNBC using a strict definition (mutations in 5 genes or *BRCA1* methylation), and 23% with a wider 20-gene panel [53]. Lenz et al. (2023) using genomic instability scores, noted higher rates in TNBC (65%), followed by HER2-positive (40%), luminal B (25%), and luminal A (15%) [69]. Chien-Feng Li et al. (2022) found 55% HRD prevalence in Taiwanese TNBC using genome-wide LOH [85]. According to Panagopoulou et al. (2024), 60–65% HRD prevalence in TNBC was found using liquid biopsy-based *BRCA1/2* methylation analysis [86]. Li et al. (2025) explored how *ZNF251* haploinsufficiency might reduce HRD prevalence by 5–10% in *BRCA1*-mutated breast cancers [87]. Galland et al. (2023) estimated 30–35% HRD in early breast cancer, rising to 55–60% in TNBC [56]. Ballot et al. (2022) identified 25–30% HRD in *BRCA*-proficient, ER-positive/HER2-negative breast cancers [77]. Zhang et al. (2022) reported 50–60% HRD in early-stage TNBC, correlating with platinum response [71]. Xiao Liu et al. (2022) observed 47% overall HRD in a Chinese cohort, with TNBC at 68% [73]. Jeon et al. (2025) detailed HRDsig positivity rates across stages and subtypes, finding it in 16.5% of HRR wild-type cancers [52]. Walens et al. (2022) found U-HRD profiles in 39.7% of CBCS and 29.3% of TCGA breast tumors, enriched in Black patients and linked to *TP53* mutant-like status [88]. Furlanetto et al. (2022) estimated 50–55% HRD in metastatic TNBC and 20–25% in non-TNBC metastatic cases [89]. Bergstrom et al. (2024) used AI-based histologic analysis to estimate HRD at 40–45% in breast cancers [90]. Methodological differences and cohort size influence estimates, with multi-scale approaches enhancing detection sensitivity (Section 10).

**Table 5:** Genetic and epigenetic drivers of HRD.

Mechanism	Cancer Type	Key Studies	Notes
Germline *BRCA1*/*BRCA2* Mutations	Ovarian, Breast	TCGA (2011) [57], Nakamura et al. (2025) [39]	Germline mutations drive ~19% of HGSOC and 10–15% of breast cancer HRD; regional variations in Japanese cohorts [65].
Somatic *BRCA1*/*BRCA2* Mutations	Ovarian, Breast	Andrikopoulou et al. (2022) [58], Batalini et al. (2023) [53]	Somatic mutations prominent in ~3.5% of sporadic TNBC; enhance PARPi response [73].
*BRCA1* Hypermethylation	Ovarian, Breast	TCGA (2011), Panagopoulou et al. (2024) [86]	Silences *BRCA1* expression; detected via liquid biopsy; prevalent in TNBC (60–65%) [74].
Non-*BRCA* HRR Genes (e.g., *RAD51C*/*RAD51D*, *PALB2*)	Ovarian, Breast	Torres-Esquius et al. (2024) [84], Jacobson et al. (2023) [70]	Significant in *RAD51D*-associated breast cancers; contribute to 9–20% of HRD [66].
RNA Methylation (m6A/m5C)	Ovarian, Breast	Zhao et al. (2023), Wei et al. (2021), Wu et al. (2024) [7,8,29]	Regulates HR gene expression via *METTL3*, *ALKBH5*, *IGF2BP2*; promotes R-loop accumulation [20].
CNVs and SVs	Ovarian	Min Wang et al. (2023) [61]	Contributes to HRD in HGSOC via structural variations.

Notes: Focuses on mechanistic drivers of HRD, referencing Section 4 for biomarker details. Non-*BRCA* and epigenetic factors, including m6A/m5C dysregulation, are critical for comprehensive HRD detection.

**Table 6:** Regional and population-specific variations.

Population	Cancer Type	HRD Prevalence	Key Studies	Notes
Chinese	Ovarian	~52%	Min Wang et al., 2023 [61]	CNVs contribute significantly
Chinese	Breast Cancer (TNBC)	68%	Xiao Liu et al., 2022 [73]	High burden in TNBC
Japanese	Breast	20% (*BRCA1/2*-linked)	Fujisawa et al., 2025 [65]	Broader markers increase TNBC rates
Japanese	Breast (TNBC)	50–60%	Kaneyasu et al. (2020) [83]	Includes *PALB2*, *BARD1*, *BLM*, *and ATM.*
Taiwanese	Breast (TNBC)	55%	Chien-Feng Li et al., 2022 [73]	Genome-wide LOH-based
Malaysian	Breast (TNBC)	32% (41/113)	Pan, JW et al., 2024 [39]	NanoString-based HRD200 Classifier
East Asian	Breast	12–13%	Ren et al. (2025) [5]	Biallelic *BRCA1*/*BRCA2*, *RAD51C*, *RAD51D*, *PPP2R2A*, *TP53* alterations

Notes: Summarizes regional variations, highlighting methodological differences affecting estimates. Chinese Cohorts: The 52% HRD prevalence in ovarian cancer reflects a broad assessment, while the 68% in TNBC suggests a higher burden in this aggressive subtype. Japanese Cohorts: The lower 10–20% HRD prevalence in breast cancer is tied to *BRCA1*/*BRCA2* mutations alone, but broader markers (e.g., *BRIP1*, *BARD1*) elevate TNBC estimates to 50–60%, indicating variability in detection methods. Taiwanese Cohorts: The 55% HRD prevalence in TNBC, based on genome-wide LOH, aligns closely with Japanese TNBC estimates using broader markers, suggesting methodological consistency.

Key Points: Section 7—HRD in Breast Cancer

- TNBC exhibits the highest HRD prevalence (50–70%), followed by HER2-positive (30–40%) and luminal subtypes (15–35%).
- Epigenetic silencing of *BRCA2*/*RAD51C* drives ~30% HRD in male breast cancer.
- Liquid biopsy-based *BRCA1*/*BRCA2* methylation analysis enhances TNBC detection (60–65%).
- *ZNF251* haploinsufficiency may reduce HRD prevalence by 5–10% in *BRCA1*-mutated breast cancers.

## 8. HRD Detection Methodologies—Present Diagnostic Methods

HRD-positive tumors are highly responsive to PARPi and platinum-based therapies due to their impaired DNA repair capabilities, making accurate HRD detection critical for treatment stratification in ovarian and breast cancers. HRD detection involves genomic assays identifying causative mutations and resultant genomic scars, as well as functional assays assessing real-time DNA repair capacity. These methods, detailed in Section 8.1 and Section 8.2 guide therapeutic decisions, with clinical implications discussed in Section 9. Integrating epigenetic analysis (e.g., *BRCA1/RAD51C* methylation) and biallelic inactivation reporting enhances detection accuracy, broadening PARPi eligibility across cancer types, including pancreatic and prostate cancers [34,35,36,67,91].

### 8.1. HRD Estimation via Functional Assays

Functional assays measure real-time DNA repair capacity, offering direct insights into HR pathway defects. The RAD51 foci formation assay, a cornerstone method, quantifies subnuclear RAD51 foci formed during S/G2 phases in response to DNA DSBs [92]. RAD51, recruited by the BRCA1/PALB2/BRCA2 complex, facilitates strand invasion and repair, with low foci counts indicating HRD and predicting sensitivity to PARPi and platinum therapies [92]. The RECAP test, based on RAD51 foci, evaluates HRD in patient-derived samples, showing promise for predicting therapeutic outcomes [92]. Compadre et al. (2023) demonstrated that low RAD51 foci (RAD51 score ≤10%) correlate with platinum response in ovarian cancer [93]. In the GeparOLA trial (n = 97), Villacampa et al. (2025) confirmed RAD51 testing’s utility in early HER2-negative breast cancer, with 80% patients (72/90) exhibited low RAD51 foci (<10%), with 66.7% (48/72) achieving pathological complete response (pCR) to PARPi or platinum therapies [94]. Similarly, Llop-Guevara et al. (2021) reported 66% pCR in untreated TNBC with low RAD51 foci (RAD51 score ≤10%) in the GeparSixto trial [95]. Analyses using TCGA data have shown that miR-622 is involved in the functionality of the HR pathway, with high tumor expression linked to increased risk of progression and death in HGSOC, independent of *BRCA* mutations. In the HERO trial, Leman et al. (2025) evaluated the concordance or complementarity of serum miR-622 (using a predefined cutoff of 77 copies/µL) with the GIScar HRD assay and explored its role in a composite score with metabolic biomarkers to predict HRD status in HGSOC. The study demonstrated that integrating serum miR-622 with the GIScar assay enhanced the prediction of platinum sensitivity, achieving a sensitivity of approximately 85% for detecting HRD, with the composite score showing improved discriminatory power for identifying platinum-sensitive tumors compared to either biomarker alone [12].

Complementary markers, such as γH2AX and 53BP1 foci, provide insights into DSB detection and repair pathway choice. γH2AX marks DSB sites, validating damage recognition, while 53BP1 promotes NHEJ, competing with HRR [96,97]. Bártová et al. (2019) noted 53BP1′s role in modulating chromatin structure and repair fidelity, contributing to HRD phenotypes when overexpressed [98]. Talens et al. (2024) found that RAD51 recruitment, but not replication fork stability, correlates with PARPi response in ovarian cancer xenografts [99]. Pae et al. (2024) showed PLK1 overexpression suppresses RAD51 foci, enhancing HRD and PARPi sensitivity [40].

Despite their potential, functional assays face challenges in clinical adoption. Technical inconsistencies, such as variability in detection methods (immunofluorescence vs. immunohistochemistry) and quantification protocols, lead to inconsistent results. The assay’s reliance on S/G2 phase activity results in a ~20% failure rate in low-proliferation tumors [95]. Molecular alterations, like *ATM* mutations, may retain RAD51 foci despite HRD, complicating interpretation [100]. Lee et al. (2023) introduced a high-throughput activity-based test for real-time HRD assessment across cancer types, but it remains experimental [101]. Doig et al. (2023) emphasized the need for standardized protocols to reduce variability [102]. Functional assays offer ~80% sensitivity but require integration with genomic assays, methylation analysis, and biallelic reporting for comprehensive HRD detection.

Key Points: Section 8.1–HRD Estimation via Functional Assays

- RAD51 foci assays quantify real-time HRD, predicting PARPi and platinum response
- GIScar assay, integrating miR-622, achieves 85% sensitivity in HGSOC [12].
- Low RAD51 foci correlate with 66.7–70% pCR in early HER2-negative breast cancer].
- Technical variability and ~20% failure rate in low-proliferation tumors limit adoption.

### 8.2. HRD Detection via Genomic Features

Genomic assays detect HRD by identifying causative mutations and genomic scars using tissue or liquid biopsies. Formalin-fixed, paraffin-embedded (FFPE) or fresh-frozen tissue biopsies are the gold standard due to their reliability in capturing genomic alterations [58]. Standardized sample preparation is critical to ensure DNA quality for mutation and epigenetic detection [91]. Marconato et al. (2025) advocated standardizing preparation for assays like Myriad myChoice CDx, SOPHiA DDM, and AmoyDx [15].

#### 8.2.1. Genomic Assays

The HRD score (GIS/HRDsum), an unweighted sum of LOH, TAI, and LST, quantifies genomic instability, predicting platinum response. However, static genomic scars may not reflect dynamic HRD status, as reversion mutations can restore HRR function, reducing PARPi efficacy [91]. WES-based assays, while cost-effective, may miss 5–10% of large *BRCA1/BRCA2* deletions due to limited coverage of intronic regions, whereas WGS captures these structural variants with high correlation (~0.95) to SNP array-based scores, serving as the gold standard for HRD detection [103].

Dong et al. (2024) reported high HRD scores correlate with improved overall survival (OS) and progression-free survival (PFS) in ovarian serous carcinoma but reduced OS in basal subtype breast carcinoma [91]. Pipeline choice (e.g., Sequenza vs. PureCN) affects prevalence estimates, highlighting the need for harmonized bioinformatics [91]. Myriad myChoice CDx (GIS ≥ 42) achieves 89% sensitivity for *BRCA1/BRCA2*-related HRD but may miss non-*BRCA* cases (e.g., *RAD51C*), while FoundationOne CDx (gLOH ≥16%) offers 85% specificity for LOH detection but struggles with heterozygous deletions (e.g., *ATM*) [61,75]. GSS, calculated from NGS or SNP array data, complements WES by detecting HRD through global genomic instability, identifying cases missed due to large BRCA deletions. Biallelic inactivation and *BRCA1/RAD51C* methylation analysis enhance non-*BRCA* HRD detection. Kang et al. (2024) validated Oncomine’s 95.8% accuracy in HGSOC [51]. Low-pass WGS assays (DirectHRD, GSscan, shallowHRD, AcornHRD) achieve high accuracy (AUC 0.980–0.997) in breast cancer [49,50]. Abbasi et al. (2025) and Lim and Ju (2025) introduced HRProfiler, a WES-based tool with AUC >0.90 [10,11]. Sahajpal et al. (2023) showed OGM detects 70.8% additional HRD signatures (e.g., translocations) [13]. Magadeeva et al. (2023) confirmed aCGH’s 77% sensitivity in ovarian cancer [14].

Genomic scars, such as SBS3, SBS39, SBS8, ID6 (microhomology-mediated deletions), CN17 (LOH segments), and SV3 (tandem duplications), reveal HRD-driven instability [13,48,104]. SBS39 shows stronger correlation with HRD-related genes than SBS3 (correlation coefficient 0.9 vs. 0.7) [104]. The NanoString-based HRD200 classifier, trained on 217 genes from the MyBrCa dataset (n = 129, Malaysian patients) and validated across TCGA, METABRIC, and Nik-Zainal cohorts, enhances HRD detection in TNBC [70]. Table 7 summarizes these signatures.

**Table 7:** HRD Score and COSMIC mutational signatures.

Feature	Description	Association with HRD
HRD-Score (GIS/HRDsum)	Unweighted sum of LOH, TAI, LST	Predicts platinum response; limited by dynamic HRD status due to reversion mutations [91]
SBS3 (COSMIC Signature 3)	96 SBS types (C>A, C>G, C>T, T>A, T>C, T>G); linked to indels and rearrangements	Enriched in germline and somatic*BRCA1/BRCA2* mutations [105,106]
SBS39	Specific SBS pattern	Stronger correlation with HRD genes than SBS3; potential HRD indicator [104]
SBS8	C>A, C>T, T>A substitutions	Non-canonical signature; Not HRD specific; Likely HRD-associated in *BRCA1/BRCA2-*deficient tumors [106]
ID6 (Indel 6)	≥5 bp deletions with microhomology	Correlated with SBS3 and HRD; BRCA2-type HRD [48,70]
ID8 (Indel 8)	≥5 bp deletions with microhomology	Linked to NHEJ, not directly HRD-specific [45]
DBS13 (Double Base Substitution 13)	TC>NN dinucleotide mutations	Indirect HRD association with SBS3, not directly HRD-specific [45,107,108]
CN17 (Copy Number 17)	LOH segments (copy number 2–4), heterozygous segments (copy number 3–8), 1–40 Mb	Strongly linked with HRD; Found in biallelic HR gene loss (*BRCA1/BRCA2*, *PALB2*) [45,107,108]
SV3/RS3 (Structural Variation 3/Rearrangement Signature 3)	Tandem duplications of 1–100 kb	Enriched in *BRCA1*-mutated tumors; referred as “Rearrangement Signature RS3” [41]

Notes: COSMIC Classification: Mutational signatures are Single Base Substitutions (SBSs), Double Base Substitutions (DBSs), Indels (ID), Copy Number Variations (CN), Structural Variations (SV), and RNA Single Base Substitutions (RNA-SBS) (Catalogue of Somatic Mutations in Cancer, v3.4). SBS39: Ding et al. (2024) reported that, in COSMIC v3.4, SBS3 has a weaker association with HRD, while SBS39 shows a stronger association with mutations in HRD-related genes, supporting its classification as an HRD-specific mutational signature [104]. Clinical Relevance: Genomic scars and mutational signatures reflect historical DNA repair defects but may not align with current HRD status due to dynamic changes (e.g., reversion mutations). New assays like DirectHRD and OGM enhance detection of ID6 and CN17/SV3, respectively [13,48]. Table 8 summarizes Next-generation sequencing based HRD detection methods.

**Table 8:** Next-generation sequencing based HRD detection methods.

Method	Description		HRD Criteria	Key Features and Advantages		Limitations
Targeted Panels	Sequence HRR genes (2–700).		GIS ≥ 42, gLOH ≥ 16%).	Cost-effective (~USD 1000), fast, hybrid capture preferred over amplicon-based for detecting large indels; off-the-shelf or custom panels.		Limited to targeted regions; amplicon risks misdiagnosis.
Shallow WGS (sWGS)	Low-pass WGS.		LGAs > 20 (shallowHRD); SeqOne score > 50%.	Broad coverage, cheaper than WGS, Detects CNAs accurately, uses tools like shallowHRD, ChosenHRDw, AcornHRD.		Low cellularity, GC bias.
Whole Exome Sequencing (WES)	coding regions only.		HRDetect > 70%; CHORD > 0.5.	Balances cost, data volume, uses tools like HRDetect, CHORD.		Misses non-coding alterations; limited detection of large *BRCA* deletions (5–10%) [105].
Whole Genome Sequencing (WGS)	entire genome (coding + non-coding).		e.g., HRDetect > 70% (breast), >99% (ovarian); CHORD > 0.84 (ovarian).	Comprehensive detection, gold standard for mutational signatures; uses HRDetect, CHORD.		Expensive (USD 5000–USD 10,000), data-intensive, hard to implement.
**Commercial NGS Tests**						
**Test**	**Provider**	**Sample**	**Key Features**	**HRD Criteria**	**FDA** **Approval**	**Notes**
MyChoice^®^ CDx	Myriad Genetics	FFPE	GIS (LOH + LST + TAI), *BRCA1/2* mutations; optional 13 HRR genes.	GIS ≥ 42 or *BRCA1/2* mutation.	Yes	Threshold varies (e.g., ≥33 for veliparib).
BRACAnalysis CDx^®^	Myriad Genetics	Blood (EDTA)	Germline *BRCA1/2* mutations only.	Deleterious *BRCA1/2* mutation.	Yes	No HRD score; misses somatic mutations.
FoundationOne^®^ CDx	Foundation Medicine	FFPE	324 genes, gLOH, *BRCA* status, MSI, TMB.	gLOH ≥ 16% or *BRCA* mutation.	Yes	Requires ≥ 35% tumor cells; misses some large rearrangements.
FoundationOne^®^ Liquid CDx	Foundation Medicine	cfDNA (plasma)	311 genes, *BRCA1/2/ATM* mutations.	*BRCA/ATM* mutations at specific VAF thresholds.	Yes	Liquid biopsy option; VAF-based criteria.
Tempus HRD	Tempus Labs	FFPE	gLOH, *BRCA1/2* LOH; RNA model option.	gLOH ≥ 21% (breast), ≥17% (ovarian), or *BRCA* mutations; RNA score ≥ 50.	No	Dynamic phenotype via RNA; discrepancies with CHORD.
CancerPrecision^®^	CeGaT	FFPE or blood	HRD score from LOH, LST, TAI; *BRCA* variants.	HRD score ≥ 30 or *BRCA* mutation.	No	Includes molecular tumor board suggestions.
MI Exome™	Caris Life Sciences	FFPE	22,000 genes, gLOH, LST; *BRCA* status.	gLOH + LST high or *BRCA* mutation.	No	Limited to specific PARPi indications; not universally available.
AmoyDx^®^ HRD Focus	Amoy Diagnostics	FFPE	Genomic Scar Score (GSS) via CNVs, *BRCA1/2* status.	GSS ≥ 50 or *BRCA* mutation.	No	High concordance with MyChoice^®^ (87.8%), Validated by Kang et al. (2024) [40].
TruSight™ Oncology 500 HRD	Illumina	FFPE	523 genes, GIS (LOH, LST, TAI), *BRCA* rearrangements.	GIS-based; high concordance with MyChoice^®^.	No	Requires ≥ 32% tumor content; not available in Japan.
SeqOne HRD Solution	SeqOne Genomics	FFPE	*BRCA* status + sWGS-based score (LGAs, LPC, *CCNE1/RAD51B*).	Score > 50% or *BRCA* mutation.	No	95% concordance with MyChoice^®^; flexible workflow.
SOPHiA DDM™ HRD	SOPHiA Genetics	FFPE	28 HRR genes + sWGS; Genomic Integrity Index (GII).	GII ≥ 0 or *BRCA* mutation.	No	94.5% concordance with MyChoice^®^; deep learning-based, Validated by Kang et al. (2024) [40].

#### 8.2.2. Companion Diagnostic Assays

Companion diagnostic (CDx) assays, such as BRACAnalysis CDx and Myriad myChoice CDx, identify HRD-positive patients for PARPi therapy. BRACAnalysis CDx uses PCR and Sanger sequencing to detect germline *BRCA1/BRCA2* mutations with >95% specificity but misses somatic and epigenetic alterations [24,109]. Myriad myChoice CDx employs NGS to analyze *BRCA1/BRCA2* mutations and HRD scores (LOH, TAI, LST) in FFPE tissue, with a score ≥42 indicating HRD positivity [61,75,81,109]. Caris HRD, using WES (2.7M SNPs), calculates Genomic Scar Score (GSS; LOH + LST) with a threshold of ≥42, achieving >97% concordance with Myriad myChoice CDx in ovarian cancer and extending validation to breast, prostate, and pancreatic cancers [64,69,75,76,81,84]. Torres-Esquius et al. (2024) reported 70–80% of *RAD51C/RAD51D*-mutated tumors exceed this threshold [84]. Engebrethsen et al. (2023) confirmed its utility in luminal breast cancer (~25% HRD-positive) [76], and Yndestad et al. (2023) validated it in HR+/HER2- (15–20%) and HER2-positive (30–35%) breast cancers [75]. Feng et al. (2023) aligned GSS with HRD scores [81]. Lenz et al. (2023) noted subtype-specific genomic instability scores (GIS) complement HRD scores in breast cancers [69]. Jacobson et al. (2023) and Lim et al. (2023) proposed enhancements with multi-scale genomic features and machine learning-based signatures for refined HRD identification [70,72]. Batalini et al. (2023) reported high HRD scores predict PARPi response in somatic *BRCA1/BRCA2* and germline *PALB2* mutations [54]. Zhang et al. (2022) confirmed HRD status as a strongpredictor of platinum response in early-stage TNBC [71]. André et al. (2020) suggested including epigenetic markers for male breast cancer [78]. Min Wang et al. (2023) reported 52% HRD positivity in Chinese HGSOC, enhanced by CNVs [61]. Weichert et al. (2022) showed 92% positive percent agreement (PPA) for *BRCA1/BRCA2* and 87% for HRD scores with NGS harmonization [64]. Wu et al. (2020) developed an HRD scoring algorithm based on TAI, LST, and LOH, which, with a cutoff of 42, identified BRCA-deficient cases with sensitivities of 94.1% in ovarian cancer, 60% in breast cancer, and 82% overall [65]. Pfarr et al. (2024) demonstrated a median concordance of 94% (range: 0–100%) in HRD classification between Myriad myChoice and a suite of alternative assays—including AmoyDx GSS v1.1.1, CytoSNP, OncoScan (using two algorithms), Illumina TSO500 HRD, Qiagen QIAseq HRD panel, whole genome sequencing (WGS), and Agilent NOGGO GISv1. This high concordance, observed across multiple platforms, supports the analytic robustness of HRD detection beyond the Myriad myChoice assay [110]. Similarly, Fountzilas et al. (2023) evaluated HRD status agreement in ovarian cancer, reporting an overall percent agreement (OPA) of 88.6% for the AmoyDx HRD Focus Panel and 77.5% for OncoScan™ when compared to Myriad myChoice. Importantly, the AmoyDx assay showed a 0% false-negative rate but a 31.6% false-positive rate relative to Myriad myChoice, indicating strong yet imperfect alignment, and reinforcing the need for ongoing cross-validation between platforms [111]. Timms et al. (2020) showed that the Myriad myChoice HRD assay—combining BRCA1/2 mutation status and multiple genomic instability measures—identifies significantly more HRD-positive ovarian cancer patients than either percent loss of heterozygosity (%LOH) testing or an 11-gene panel. Up to 61% of patients flagged as HRD-positive by myChoice would be missed by these simpler methods [109]. Jiao et al. (2019) introduced the ASGAD algorithm, achieving 93% PARPi response accuracy [112]. Li et al. (2025) noted *ZNF251* haploinsufficiency may cause false negatives, suggesting additional markers [87]. Kang et al. (2024) validated Oncomine’s utility [51]. Table 9 summarizes Myriad myChoice CDx and Caris HRD applications.

#### 8.2.3. FoundationOne CDx

FoundationOne CDx (F1CDx) profiles tumor tissue or circulating tumor DNA (ctDNA) using NGS, detecting mutations, CNAs, and rearrangements across 324 genes, supporting PARPi eligibility in breast and ovarian cancers [115]. It provides data on microsatellite instability (MSI), tumor mutational burden (TMB), and HRD status (somatic *BRCA*-positive and/or LOH-high ≥16%), but struggles with heterozygous deletions (e.g., *ATM*). Its limitations in detecting large *BRCA* rearrangements are mitigated by complementary use with Caris HRD’s WES-based GSS, which offers LOH accuracy comparable to WGS [113,114]. Chien-Feng Li et al. (2023) demonstrated that genome-wide LOH quantification using the standard F1CDx threshold (LOH ≥16%) effectively identifies HRD-positive TNBC cases, showing high concordance with F1CDx and supporting its use as a cost-effective clinical alternative [81]. Quesada et al. (2023) confirmed that both F1CDx and Myriad myChoice CDx identify 50–51% of cases as HRD-positive using BRCA1/2 mutation and LOH criteria, and highlighted the robust cross-platform agreement for establishing PARP inhibitor eligibility [60]. Marconato et al. (2025) noted its broader genomic profiling but limited HRD specificity compared to dedicated assays like SOPHiA DDM, which are optimized specifically for detecting HRD-related genomic scars and biallelic inactivation [15]. Table 10 highlights F1CDx’s strengths (85% specificity for LOH) and limitations (60% sensitivity for non-*BRCA* HRD), suggesting complementary use with Myriad, Caris, methylation analysis, and biallelic reporting [29,33,34,35,36,73,107].

#### 8.2.4. Advanced Genomic Tools

HRDetect, a WGS-based tool, uses machine learning to analyze six mutational signatures, including Signature 3 and microhomology-mediated indels, offering high sensitivity (>90%) for detecting “BRCAness” in sporadic cancers [105]. WGS-based methods like HRDetect and CHORD outperform WES and array-based assays in detecting structural variants and tandem duplications, with correlation coefficients ~0.95 to SNP arrays [103]. Lim et al. (2023) reported 60–70% HRD prevalence in TNBC using machine learning-based signatures [72]. Nakamura et al. (2025) highlighted NGS panels targeting *BRIP1*, *BARD1*, and *RAD51* paralogs for improved sensitivity in non-*BRCA* HRD [39]. Xiao Liu et al. (2022) showed HRDetect’s utility in Chinese breast cancer patients [48]. Andrews et al. (2024) noted variable non-*BRCA* HRD detection across 20 assays (correlation coefficients 0.4–0.9), emphasizing harmonization needs [63]. Abbasi et al. (2025) and Lim and Ju (2025) introduced HRProfiler (AUC >0.90) [10,11]. Sahajpal et al. (2023) showed OGM detects 70.8% additional HRD signatures [13]. Magadeeva et al. (2023) confirmed aCGH’s 77% sensitivity for HRD in ovarian cancer [14]. HRDetect’s high cost (USD 5000–USD 10,000) limits routine use, requiring integration with functional assays, methylation analysis, and biallelic reporting.

#### 8.2.5. Integration of Detection Methods

Emerging approaches, such as liquid biopsy-based HRD testing using circulating tumor DNA (cfDNA) and shallow WGS (1×–2× coverage), achieve high sensitivity (90% at 1% tumor fraction) and cost-effectiveness, enabling non-invasive monitoring and population-scale screening [86]. AI-driven tools like DirectHRD and optimized HRDscar detect subtle microhomology-mediated deletions, improving non-*BRCA* HRD detection in low-purity samples [36,91]. Multi-omics integration, combining transcriptomic signatures with WGS/WES, enhances dynamic HRD assessment, particularly for ambiguous genomic scars [91]. Zhang et al. (2025) proposed MODeepHRD, a multi-omics deep-learning platform for the prediction of homologous recombination deficiency (HRD) in gynecological cancers, but it does not include epitranscriptomics (RNA modification profiling) as part of its input or analytic approach [116].

Key Points: Section 8.2—HRD Detection via Genomic Features

- Myriad myChoice CDx and FoundationOne CDx are standards, with Caris HRD offering >97% concordance via WES-based GSS.
- RAD51 foci and GIScar assays assess dynamic HRD status with 85% accuracy
- Low-pass WGS (DirectHRD, AcornHRD) offers cost-effective detection (AUC 0.980–0.997).
- OGM and aCGH capture 70.8–77% of HRD signatures, enhancing sensitivity

## 9. Challenges in Clinical Implementation

The clinical implementation of HRD detection is hindered by assay variability, high costs, limited tissue availability, and disparities in access, impacting consistent classification and equitable therapeutic decision-making for PARPi and platinum-based therapies in ovarian and breast cancers. Recent consensus recommendations highlight the need for standardized assay design, including gene panels (*BRCA1*, *BRCA2*, *PALB2*, *RAD51C*, *RAD51D*), scar definitions (LOH, LST, TAI), and robust validation with defined specimen requirements to ensure analytical and clinical validity [117]. Efforts to standardize HRD detection with assays like Myriad myChoice CDx and FoundationOne CDx continue, but challenges in defining consistent thresholds persist [61,91,102,110,111]. In ovarian cancer, concordance for *BRCA1/BRCA2*-related HRD is high, but non-*BRCA* HRD detection (e.g., *RAD51C* mutations) varies significantly (60–70% agreement), necessitating standardized scoring and validation to ensure equitable patient outcomes [63]. Discrepancies between Myriad myChoice CDx (GIS ≥ 42, 89% sensitivity for *BRCA1/BRCA2*) and FoundationOne CDx (gLOH ≥ 16%, 85% specificity for LOH) can miss up to 20% of non-*BRCA* HRD cases, potentially excluding PARPi-eligible patients and reducing survival benefits [61]. WES-based assays, such as Caris HRD, miss 5–10% of large *BRCA* deletions, requiring complementary GSS to capture global genomic instability [103,113,114]. Variability in non-*BRCA* HRD detection across platforms like SOPHiA DDM and AmoyDx further complicates consistent classification, highlighting the need for harmonized protocols [15]. Oncomine’s 95.8% concordance with SOPHiA DDM’s Genomic Integrity Index supports decentralized testing to reduce variability [51]. Integrating functional and genomic assays enhances reliability across tumor types, including pancreatic and prostate cancers [118]. Pathologists play a critical role in standardizing interpretation protocols to minimize assay variability [102]. Inconsistent detection of *BRCA1* methylation in mutation-negative cases adds complexity to HRD classification [6].

The high cost of comprehensive genomic profiling, particularly whole-genome sequencing (WGS, USD 5000–USD 10,000), and the need for high-quality tissue samples limit accessibility, especially in low-resource settings. WGS offers superior sensitivity for structural variants and mutational signatures, with ~0.95 correlation to SNP arrays, but its cost restricts routine use [103]. Cost-effective alternatives, such as low-pass WGS assays (DirectHRD, GSscan, shallowHRD, AcornHRD, ~USD 1000), achieve high accuracy (AUC 0.980–0.997) for HRD detection in breast cancer [36,37,38,39]. HRProfiler, a WES-based tool, offers comparable sensitivity (AUC > 0.90) at reduced costs [10,11]. OGM detects 70.8% additional HRD signatures (e.g., translocations), and high-resolution aCGH achieves 77% sensitivity in ovarian cancer, but OGM’s non-standardized thresholds and aCGH’s patent restrictions (~EUR 300) hinder widespread adoption [13,14]. Higher HRD prevalence in Black and Asian populations underscores the need for accessible diagnostics to address equity [5,88].

Functional assays, such as RAD51 foci formation, provide dynamic HR competency assessment but are technically challenging. The RAD51 foci assay, which quantifies subnuclear foci during S/G2 phases, correlates with PARPi and platinum response but suffers from a ~20% failure rate in low-proliferation tumors and variability in detection methods (e.g., immunofluorescence vs. immunohistochemistry) [12,92,119]. The DNA fiber assay is a powerful, microscopy-based technique for studying DNA replication fork dynamics in vitro, offering functional insights into replication fork protection, degradation, and stability—factors that critically influence chemotherapy and PARP inhibitor efficacy in ovarian and breast cancer. However, its routine clinical application is limited by technical complexity, lack of standardized protocols, low throughput, and significant operator dependence [120]. The GIScar assay, combining RAD51 foci and serum miR-622, reduces inconclusive results in HGSOC but requires broader validation [12]. Reversion mutations restoring HR function can reduce PARPi efficacy, necessitating functional assays to complement genomic approaches [9]. Resistance mechanisms, such as *SETD1A/EME1* and *SOX5* alterations, may cause false negatives in genomic assays, highlighting the need for integrated functional testing [18,19]. Liquid biopsies using circulating tumor DNA (ctDNA) enable minimally invasive monitoring, with DirectHRD achieving 90% sensitivity at 1% tumor fraction, but are limited by false negatives from low ctDNA levels and false positives from clonal hematopoiesis of indeterminate potential (CHIP) require methylation analysis improving detection in mutation-negative cases. Advanced tools like HRDetect, which uses machine learning to analyze mutational signatures (e.g., SBS39), require validation in larger cohorts (n > 1000) to confirm predictive power. Biallelic inactivation reporting enhances PARPi response prediction, particularly in *BRCA*-associated tumors (>80% biallelic rate) [54]. The Archer™ VARIANTPlex HRD workflow avoids paired normals but risks lower specificity, while OncoScan’s high-resolution SNP arrays (220,000 SNPs) require higher DNA input and paired normals. The NanoString-based HRD200 classifier, trained on 217 genes and validated across diverse cohorts, offers a promising approach for TNBC [121]. These challenges—assay variability, high costs, access disparities, and the need for detailed clinical reporting (e.g., assay limitations, HRR genes, biallelic status, methylation results)—emphasize the urgent need for harmonized protocols, cost-effective assays, and integrated functional-genomic approaches, as recommended by consensus guidelines, to ensure accurate and equitable HRD detection for optimal patient care [117].

Key Points: Section 9—Challenges in Clinical Implementation

- Assay variability (60–70% non-*BRCA* concordance) and high WGS costs (USD 5000–USD 10,000) limit HRD detection.
- WES misses 5–10% of large *BRCA* deletions, requiring GSS to enhance detection
- Liquid biopsy (cfDNA) and low-pass WGS improve accessibility, with 90% sensitivity at low tumor fractions
- Standardized thresholds, methylation analysis, and biallelic reporting are critical for equitable outcomes

## 10. Homologous Recombination Deficiency (HRD) as an Actionable Therapeutic

HRD is a pivotal biomarker in oncology, particularly for ovarian and breast cancers, expanding actionable therapeutic targets beyond *BRCA1/BRCA2* mutations to enhance patient stratification for PARPi and platinum-based therapies. HRD drives synthetic lethality by impairing HR repair, making cells vulnerable to combined inhibition of HR and BER pathways via PARPi [3]. This section integrates clinical trial data (e.g., SOLO1, OlympiAD) and real-world evidence to highlight HRD’s therapeutic potential, addressing resistance mechanisms and the need for standardized detection to ensure equitable patient access

To address the challenges of HRD detection and therapy, we propose a refined conceptual model integrating genomic (e.g., Myriad myChoice CDx, FoundationOne CDx, Caris HRD), functional (e.g., RAD51 foci, DNA fiber assay), epigenetic (e.g., *BRCA1*/*RAD51C* methylation) assays and epitranscriptomic RNA modifications/methylation (e.g., to capture the full HRD spectrum, including non-*BRCA* defects. This model leverages machine learning-based multi-omics profiling (e.g., HRProfiler, ASGAD), *BRCA1*/*RAD51C* methylation analysis, and biallelic inactivation reporting to enhance detection precision and predict PARPi response [10,11,33,34,35,36,73,84]. Novel tools like GIScar, OGM, and high-resolution aCGH improve sensitivity, while combination therapies (e.g., PARPi with *ATR* inhibitors) target resistance mechanisms [12,41,42,44,45]. Table 11 outlines its components, providing a framework for advancing precision oncology [5,10,11,12,33,34,35,36,41,42,44,45,107,108].

In HGSOC, where ~50% of cases exhibit HRD, patients show enhanced survival with platinum-based chemotherapy (e.g., cisplatin, carboplatin) and PARPi maintenance (e.g., olaparib, niraparib, rucaparib) [95,97]. Platinum agents induce DSBs via DNA crosslinking, which HR-deficient cells cannot repair, while PARPi inhibit BER, converting single-strand breaks (SSBs) into lethal DSBs during replication. Clinical trials like SOLO1 demonstrate that PARPi maintenance extends PFS by 12–36 months in HRD-positive HGSOC [122]. In *BRCA1/BRCA2*-mutated HGSOC, response rates to PARPi and platinum therapies exceed 70% in advanced stages, while HRD-positive patients with non-*BRCA* defects (e.g., *RAD51C*) achieve 60–80% objective response rates (ORR) and 12–18 months’ PFS improvement. In Chinese HGSOC patients, high HRD scores, driven by *BRCA1/BRCA2* mutations and copy number variations (CNVs), correlate with 75% response rates [61]. Fiegl et al. (2024) showed *BRCA1* methylation predicts 99% platinum sensitivity in HGSOC, identifying additional HRD-positive cases [6]. Leman et al. (2025) validated the GIScar assay, combining RAD51 foci and serum miR-622, achieving 85% accuracy for predicting PARPi/platinum response in HGSOC [12]. Kang et al. (2024) confirmed Oncomine’s 95.8% accuracy for identifying HRD-positive HGSOC eligible for niraparib [51]. Ren et al. (2025) reported biallelic alterations in *BRCA1/BRCA2*, *RAD51C*, *RAD51D*, *PPP2R2A*, and *TP53* in Asian cohorts predict PARPi response, broadening therapeutic applicability [5]. The ASGAD algorithm integrates *BRCA1/BRCA2* mutations and genomic scars to predict PARPi response with 93% accuracy [112]. Jiang et al. (2025) extended HRD’s prognostic and predictive value to gastrointestinal cancers, showing high HRD scores correlate with poorer survival but better response to platinum and PARPi in BRCA-wild-type cases [123]. However, variable detection of non-*BRCA* HRD across assays may exclude PARPi-eligible patients, underscoring the need for improved tools [63]. Biallelic inactivation reporting and *BRCA1/RAD51C* methylation analysis enhance identification of HRD-positive cases.

In breast cancer, particularly TNBC, HRD predicts responsiveness to platinum agents and PARPi like talazoparib, especially in *BRCA1/BRCA2*-mutated cases [124]. Somatic *BRCA1/BRCA2* mutations, though rare (~3.5%) in sporadic TNBC, confer significant sensitivity to DNA-damaging therapies [125]. “BRCAness”—HRD in sporadic cancers without *BRCA1/BRCA2* mutations—extends actionable targets to genes like *ATM*, *RAD51C*, and *PALB2* [95]. *RAD51C/RAD51D*-mutated breast and ovarian cancers show HRD profiles predictive of PARPi response. High GSS or GIS in TNBC and luminal subtypes correlate with 60–70% PARPi response rates [69,70]. HRD also predicts sensitivity in luminal and HER2-positive subtypes with *BRCA1/BRCA2*, *ATM*, *BRIP1*, or *BARD1* defects [75,76]. In Chinese TNBC patients, *BRCA1/BRCA2*-associated mutational signatures strongly predict PARPi and platinum efficacy. Richters et al. (2025) reported 15.8% of TNBC cases with *BRCA1/BRCA2* tumor pathogenic variants achieve 64.3% pCR to carboplatin-based therapy [126]. Jeon et al. (2025) identified 16% of non-*BRCA* breast cancers as HRD-positive, expanding PARPi candidates [52]. Abbasi et al. (2025) and Lim and Ju (2025) demonstrated HRProfiler’s ability to identify HRD in non-*BRCA* tumors (AUC >0.90) [10,11]. Marconato et al. (2025) noted HRD testing increases PARPi eligibility to ~50% in ovarian cancer [15]. Pae et al. (2024) identified PLK1 overexpression as an HRD driver enhancing PARPi sensitivity by suppressing HR [40]. Wu et al. (2024) showed m6A/m5C dysregulation increases PARPi sensitivity via R-loop accumulation [29]. Conversely, *ZNF251* haploinsufficiency in *BRCA1*-mutated breast cancer may reduce PARPi sensitivity by restoring HR function [87]. Resistance mechanisms, such as *SETD1A/EME1* downregulation and *SOX5* overexpression, reduce PARPi efficacy, necessitating combination therapies (e.g., with ATR inhibitors) [18,19]. The Archer™ VARIANTPlex HRD workflow, using ASCN entropy and NHEJ metrics, and OncoScan’s high-resolution SNP arrays enhance therapeutic stratification by integrating DNA/RNA methylation [16,115]. Herzog et al. (2023) highlighted HRD’s role in expanding PARPi indications to pancreatic and prostate cancers, with *BRCA2*-mutated cases showing 7.2–8.1 months’ PFS [118]. Caris HRD’s WES-based GSS (≥42) predicts PARPi benefit across ovarian, breast, prostate, and pancreatic cancers, with a hazard ratio of 0.27 for OS (*p* < 0.001) [113,114]. Addressing resistance challenges (Section 14) and standardizing detection, including biallelic and methylation status, are critical for equitable therapeutic access [34,35,36,37,74].

Key Points: Section 10—HRD as an Actionable Therapeutic

- HRD predicts 60–80% ORR to PARPi in HGSOC and 60% in TNBC, extending PFS by 12–36 months
- *BRCA1* methylation and *RAD51C*/*RAD51D* mutations expand PARPi eligibility
- Caris HRD’s GSS (≥42) enhances detection in multiple cancers, predicting improved OS
- Combination therapies (e.g., PARPi with ATR inhibitors) address resistance mechanisms

## 11. PARP Inhibitors

PARPi exploit synthetic lethality in HRD-positive cancers by trapping PARP1/PARP2 enzymes at SSB sites, converting them into cytotoxic DSBs during replication [3]. In HR-proficient cells, DSBs are repaired via HR, but HR-deficient cells accumulate unrepaired DSBs, leading to cell death. *BRCA1/BRCA2* and other HR proteins repair DSBs, while PARP enzymes manage SSBs via BER. PARPi disrupt this balance, amplifying DNA damage in HRD cells. ATM, activated by DSBs through the MRN complex, supports repair and checkpoint signaling; cells lacking ATM or NBS1 mimic *BRCA1/BRCA2*-deficient sensitivity to PARPi [9]. HRDsig identifies 82% of *BRCA1/BRCA2* or *PALB2*-mutated tumors, enhancing PARPi candidate selection [52].

Replication fork protection, as quantified by fiber length after dual labeling and replication stress, serves as a surrogate marker for sensitivity to platinum and PARP inhibitor therapies—where loss of fork protection predicts increased drug sensitivity [120]. Fork degradation, observed as shortened labeled DNA tracts in the assay, is indicative of HRD and can help predict therapeutic response to DNA-damaging agents [127]. Furthermore, examining fork stability after targeted manipulation (such as assessing the effects of nucleases like MRE11 or proteins like FANCD2) provides mechanistic insight into resistance pathways, revealing how tumors may acquire resistance to platinum and PARPi therapies. Together, these parameters functionally characterize the DNA replication stress response and are valuable for predicting both initial response and the emergence of therapeutic resistance in ovarian and breast cancer.

In HGSOC, where ~50% of cases exhibit HRD, PARPi (olaparib, rucaparib, niraparib) are FDA-approved for advanced disease. The SOLO1 trial showed olaparib maintenance in *BRCA1/BRCA2*-mutated HGSOC extended median PFS beyond 36 months [128]. The PRIMA trial demonstrated niraparib’s benefit in HRD-positive, *BRCA*-wild-type patients, achieving 50–70% ORR and 12–18 months’ PFS improvement [129]. *RAD51C/RAD51D*-mutated cases show comparable PARPi responses, broadening eligibility [84]. In Chinese HGSOC patients, HRD-positive cases with *BRCA1/BRCA2* mutations and CNVs achieve 75% ORR [61]. Fiegl et al. (2024) reported *BRCA1* methylation predicts 99% platinum sensitivity in HGSOC, identifying additional HRD-positive cases [6]. Leman et al. (2025) validated the GIScar assay, integrating RAD51 foci and serum miR-622, with 85% accuracy for PARPi/platinum response in HGSOC [12]. Kang et al. (2024) confirmed Oncomine’s 95.8% accuracy for identifying HRD-positive HGSOC (64.4% HRD positivity) for niraparib eligibility [51]. Marconato et al. (2025) noted HRD testing expands PARPi eligibility to ~50% in ovarian cancer [15]. The ASGAD algorithm predicts PARPi response with 93% accuracy by integrating *BRCA1/BRCA2* mutations and genomic scars [112]. Ren et al. (2025) reported biallelic *BRCA1/BRCA2*, *RAD51C*, *RAD51D*, *PPP2R2A*, and *TP53* alterations in Asian cohorts enhance PARPi response prediction [5]. Wanhong He et al. (2024) identified 52 methylation-driven DNA damage-response (DDR) genes with abnormal promoter methylation and expression in ovarian cancer; among these, BRCA1, PTTG1, TTK, AURKA, CDC6, and E2F1 were significantly associated with HRD scores, while E2F1, PTTG1, and CDC6 were also linked to PARP inhibitor sensitivity [130].

In breast cancer, olaparib and talazoparib are approved for *BRCA1/BRCA2*-mutated, HER2-negative metastatic TNBC. The OlympiAD trial showed olaparib improved PFS, with 60% tumor reduction compared to standard chemotherapy [24]. In Japanese TNBC cohorts, *BRCA1/BRCA2*-mutated cases align with global response rates, with preliminary *BRIP1*-mutated efficacy under investigation [82,83]. HRD-positive luminal breast cancers with *ATM* or *BRCA1/BRCA2* defects show tumor reduction with PARPi [75,76]. High HRD scores in HR+/HER2- (15–20%) and HER2-positive (30–35%) subtypes predict PARPi benefit. High GSS in TNBC (65% HRD prevalence) and high-grade luminal subtypes correlate with 6–8 months’ median PFS. Caris HRD’s GSS (≥42) predicts PARPi benefit with a hazard ratio of 0.27 (*p* < 0.001) across multiple cancers. Real-world data report median PFS of 8.3 months (somatic *BRCA1/BRCA2*), 7.9 months (germline *PALB2*), and 6.5 months (HRD signature) with olaparib [122,128]. In Taiwanese TNBC, 55% of high-LOH cases respond to PARPi, with 7–9 months’ PFS [73]. In early-stage TNBC, HRD predicts higher pCR rates with platinum-based neoadjuvant therapy [73]. In Chinese TNBC, *BRCA1/BRCA2*-linked mutational signatures drive robust PARPi responses [73]. In male breast cancer, *BRCA2/RAD51C* hypermethylation in ~30% of cases suggests PARPi potential [78]. Jeon et al. (2025) identified 16% of non-*BRCA* breast cancers as HRD-positive, expanding PARPi candidates [52]. Richters et al. (2025) reported 15.8% of TNBC cases with *BRCA1/BRCA2* variants achieve 64.3% pCR to carboplatin-based therapy [126]. Abbasi et al. (2025) and Lim and Ju (2025) showed HRProfiler’s ability to identify non-*BRCA* HRD tumors (AUC > 0.90). Pae et al. (2024) identified PLK1 overexpression as an HRD driver enhancing PARPi sensitivity. Wu et al. (2024) showed m6A/m5C dysregulation increases PARPi sensitivity via R-loop accumulation [20]. Biallelic inactivation reporting enhances PARPi response prediction, with biallelic alterations correlating with better outcomes [34,35,36,37,74]. Hill et al. (2021) showed, using high-grade serous ovarian cancer (HGSOC) organoids, that most carboplatin-sensitive and PARPi-sensitive tumors exhibited replication fork instability, while fork protection predicted resistance to platinum but not always to PARPi. Some PARPi-resistant organoids retained fork instability but were HR proficient by RAD51 foci, emphasizing that fork status and HR status can be decoupled [131].

Platinum agents (e.g., cisplatin, carboplatin) synergize with PARPi by inducing DNA crosslinks that convert to DSBs, overwhelming HRD cells. This combination is standard in HGSOC but faces resistance from *BRCA* reversion mutations, MDR1 upregulation, or HR-independent repair (e.g., NHEJ) [9]. *SETD1A/EME1* downregulation and *SOX5* overexpression are resistance biomarkers, necessitating combination therapies with ATR inhibitors [18,19]. *ZNF251* haploinsufficiency in *BRCA1*-mutated breast cancer reduces PARPi efficacy by restoring HR function [87]. Combination strategies, such as PARPi with pembrolizumab or ATR inhibitors, target immune activation or compensatory repair to overcome resistance [129]. The Archer™ VARIANTPlex HRD workflow and OncoScan’s high-resolution SNP arrays enhance PARPi candidate selection by integrating DNA/RNA methylation and CNA/LOH assessment [16,17]. In pancreatic and prostate cancers, *BRCA2*-mutated cases achieve 7.2–8.1 months’ PFS with PARPi. Addressing resistance (Section 14) and standardizing detection with biallelic and methylation status reporting are critical for equitable therapeutic access [117].

Key Points: Section 11—PARP Inhibitors

- PARPi (olaparib, niraparib) extend PFS by >36 months in *BRCA1*/*BRCA2*-mutated HGSOC (SOLO1).
- GIScar and Oncomine achieve 85–95.8% accuracy for PARPi eligibility in HGSOC
- Caris HRD’s GSS predicts PARPi benefit across multiple cancers (HR = 0.27, *p* < 0.001)
- Combination therapies with ATR inhibitors address resistance in HRD-positive tumors

## 12. Discussion

HRD is a cornerstone biomarker in ovarian and breast cancers, driving genomic instability and shaping therapeutic strategies. Deficient HRR, primarily due to *BRCA1/BRCA2* mutations, non-*BRCA* HRR gene alterations, and epigenetic changes, influences tumor behavior and enhances sensitivity to platinum-based chemotherapy and PARPi [3,9]. This discussion synthesizes recent advancements to propose a refined conceptual model (Table 11, Section 10) integrating genomic, functional, and epigenetic assays to address non-*BRCA* HRD detection, overcome resistance, and advance precision oncology through standardized reporting and multi-omics profiling.

HRD promotes tumorigenesis by increasing genomic instability, yet it confers heightened sensitivity to DNA-damaging agents. PARPi (e.g., olaparib, niraparib, rucaparib) exploit synthetic lethality in HRD-positive tumors, achieving significant PFS gains (e.g., >36 months in *BRCA1/BRCA2*-mutated HGSOC, SOLO1 trial) [132]. However, resistance mechanisms, such as *BRCA* reversion mutations, *MDR1* upregulation, *ZNF251* haploinsufficiency, *SETD1A/EME1* downregulation, and *SOX5* overexpression, reduce PARPi efficacy, necessitating dynamic HRD assessment and combination therapies (e.g., PARPi with ATR or immune checkpoint inhibitors) [9,19,87,132]. Dong et al. (2024) reported high HRD scores correlate with improved survival in HGSOC but reduced survival in basal breast cancer, highlighting subtype-specific prognostic roles [91]. Clinical trials (e.g., SOLO1, OlympiAD, PRIMA) demonstrate robust PARPi efficacy (60–80% ORR in HGSOC, 60% in TNBC), while real-world data show variable PFS (6.5–8.3 months), underscoring the need for broader HRD detection [128,132]. Richters et al. (2025) reported 64.3% pCR to carboplatin in *BRCA1/BRCA2* tumor pathogenic variant-positive TNBC [126]. Jeon et al. (2025) identified 16% of non-*BRCA* breast cancers as HRD-positive, expanding PARPi candidates. Herzog et al. (2023) extended HRD’s therapeutic relevance to pancreatic and prostate cancers, with *BRCA2*-mutated cases achieving 7.2–8.1 months’ PFS [118].

HRD testing has evolved beyond *BRCA1/BRCA2* mutation testing to include genomic scar assays (LOH, TAI, LST), functional assays (e.g., RAD51 foci), and epigenetic markers. Andrews et al. (2024) reported 60–70% concordance for non-*BRCA* HRD across 20 assays, with up to 20% of cases missed due to assay-specific limitations, necessitating integrated approaches [63]. Fiegl et al. (2024) showed *BRCA1* methylation predicts 99% platinum sensitivity in HGSOC, identifying mutation-negative HRD cases [6]. Zhao et al. (2023) and Wei et al. (2021) highlighted m6A RNA methylation regulators (*METTL3*, *ALKBH5*, *IGF2BP2*) as HRD contributors impacting prognosis [7,8]. Wu et al. (2024) demonstrated m6A/m5C dysregulation promotes R-loop accumulation, enhancing HRD and PARPi sensitivity [29]. Leman et al. (2025) validated the GIScar assay, combining RAD51 foci and serum miR-622, with 85% accuracy for HRD detection in HGSOC [12]. Abbasi et al. (2025) and Lim and Ju (2025) introduced HRProfiler, a WES-based tool with AUC >0.90 for non-*BRCA* HRD [10,11]. Kang et al. (2024) confirmed Oncomine’s 95.8% accuracy for HRD detection in HGSOC, supporting decentralized testing [51]. Liu et al. (2023) and Pan et al. (2024) developed low-pass WGS assays (DirectHRD, GSscan, shallowHRD, AcornHRD) with AUC 0.980–0.997, improving cost-effectiveness [49,74]. Sahajpal et al. (2023) and Magadeeva et al. (2023) showed OGM and high-resolution aCGH detect 70.8% and 77% of HRD signatures, respectively [13,14]. Marconato et al. (2025) advocated integrating RAD51 foci with NGS assays to expand PARPi eligibility to ~50% in ovarian cancer [15]. Ren et al. (2025) and Walens et al. (2022) emphasized higher HRD prevalence in Asian and Black populations, necessitating equitable testing to address disparities [5,88]. Emerging liquid biopsy and multi-omics approaches, including cfDNA-based DirectHRD and transcriptomic integration, enhance dynamic HRD assessment and accessibility, particularly for early-stage and non-traditional cancers [86]. Emerging therapies, such as PARPi combined with ATR or immune checkpoint inhibitors, show preclinical promise in overcoming resistance by targeting compensatory repair pathways [133]. Pae et al. (2024) identified PLK1 overexpression as an HRD driver enhancing PARPi sensitivity, offering a novel therapeutic target [40]. Non-*BRCA* HRD detection variability and resistance mechanisms underscore the need for a unified framework (Table 11, Section 10) integrating genomic, functional, and epigenetic data.

Key Points: Section 12—Discussion

- HRD drives 60–80% ORR to PARPi in HGSOC and 60% in TNBC, with resistance from *BRCA* reversion and *SOX5* alterations.
- GIScar, HRProfiler, and low-pass WGS assays enhance non-*BRCA* HRD detection (AUC >0.90).
- Liquid biopsy and multi-omics approaches improve dynamic HRD assessment.
- Equitable testing addresses higher HRD prevalence in Asian and Black populations.

## 13. Conclusions

HRD is a transformative biomarker in personalized oncology, driving therapeutic strategies in HGSOC and TNBC, where its high prevalence (~50% in HGSOC, 50–70% in TNBC) enables targeted therapies like PARPi and platinum agents. HRD extends beyond *BRCA1/BRCA2* mutations to include non-*BRCA* genes (*RAD51C/RAD51D*, *BRIP1*, *BARD1*, *ATM*, *PALB2*), broadening actionable targets across ovarian and breast cancer subtypes, including HER2-positive (30–40%) and luminal subtypes (15–25%). GIS and GSS correlate with therapeutic responsiveness, with TNBC showing up to 70% actionable HRD. Given the complexity of HRD biomarkers, key assay features include the selection of HRR genes (*BRCA1*, *BRCA2*, *PALB2*, *RAD51C*, *RAD51D*), definitions of genomic scars (LOH, TAI, LST), mutational signatures analyzed, use of comparator samples, and tumor-specific HRD score calculations. Machine learning-based mutational profiling, such as HRProfiler, enhances non-*BRCA* HRD detection with AUC > 0.90. Epigenetic silencing, particularly *BRCA1/RAD51C* methylation, identifies additional HRD-positive cases, with *BRCA1* methylation predicting 99% platinum sensitivity in HGSOC [134]. m6A/m5C RNA methylation dysregulation (*METTL3*, *ALKBH5*, *IGF2BP2*) promotes R-loop accumulation, enhancing PARPi sensitivity and impacting prognosis [7,8,29]. Biallelic inactivation reporting improves PARPi response prediction, with >80% response rates in biallelic *BRCA*-associated tumors.

In HGSOC, PARPi (e.g., olaparib, niraparib, rucaparib) extend PFS beyond 36 months in *BRCA1/BRCA2*-mutated cases (SOLO1 trial), with 60–80% ORR in HRD-positive patients, including non-*BRCA* defects. In Chinese HGSOC, high HRD scores driven by *BRCA1/BRCA2* mutations and CNVs achieve 75% ORR. In TNBC, PARPi like talazoparib yield 60% ORR (OlympiAD trial), with real-world PFS of 6.5–8.3 months across somatic *BRCA1/BRCA2*, germline *PALB2*, and HRD signature cases. Early-stage TNBC with HRD shows superior pCR rates to platinum-based neoadjuvant therapy. In Chinese TNBC, *BRCA1/BRCA2*-linked mutational signatures drive robust PARPi responses. *RAD51C/RAD51D*-mutated cancers and *BRCA2/RAD51C* hypermethylation in male breast cancer (~30% prevalence) expand PARPi candidacy]. Herzog et al. (2023) highlighted PARPi’s role in pancreatic and prostate cancers, with *BRCA2*-mutated cases achieving 7.2–8.1 months’ PFS [118]. Richters et al. (2025) reported 64.3% pCR to carboplatin in *BRCA1/BRCA2* tumor pathogenic variant-positive TNBC [126]. Jeon et al. (2025) identified 16% of non-*BRCA* breast cancers as HRD-positive, further broadening PARPi eligibility.

Advanced detection tools enhance HRD identification. Myriad myChoice CDx and FoundationOne CDx remain standards, but HRDetect, genome-wide LOH assays (55% TNBC HRD-positive), and the GIScar assay (integrating RAD51 foci and serum miR-622, 85% accuracy in HGSOC) improve precision Oncomine achieves 95.8% accuracy for HRD in HGSOC. Low-pass WGS assays (DirectHRD, GSscan, shallowHRD, AcornHRD) offer cost-effective detection (AUC 0.980–0.997). OGM and high-resolution aCGH capture 70.8% and 77% of HRD signatures, respectively The Archer™ VARIANTPlex HRD workflow and OncoScan’s SNP arrays (220,000 SNPs) integrate DNA/RNA methylation and CNA/LOH assessment for robust stratification].

However, clinical implementation is limited by heterogeneous assay designs, lack of standardization in technical performance, and inconsistent reporting practices, as highlighted by recent AMP consensus recommendations [117]. WES-based assays miss 5–10% of large BRCA deletions, requiring complementary GSS to capture global genomic instability.

To translate HRD testing’s potential into tangible patient benefits, we propose the following roadmap for clinical implementation and research priorities, building on the conceptual model in Table 11 (Section 10) and AMP recommendations [117]:

❖ Clinical Implementation:
  ○ Harmonize assay design, including standard definitions for genomic scars (LOH, TAI, LST), mutational signatures, and gene panels (*BRCA1*, *BRCA2*, *PALB2*, *RAD51C*, *RAD51D*), to reduce variability (60–70% non-*BRCA* concordance).
  ○ Integrate biallelic inactivation and *BRCA1*/*RAD51C* methylation reporting to enhance PARPi response prediction, capturing >80% of responsive *BRCA*-associated tumors
  ○ Expand access to cost-effective diagnostics (e.g., low-pass WGS, HRProfiler, ~USD 1000) to address disparities in Asian and Black populations.
  ○ Implement robust validation processes with well-defined specimen requirements and neoplastic cellularity thresholds to ensure analytical and clinical validity [117].
  ○ Implement detailed clinical reports specifying HRR genes, assay limitations, biallelic status, and methylation results to guide provider decision-making.
❖ Research Priorities:
  ○ Refine predictive thresholds and tumor-specific cutoffs (e.g., GSS ≥ 42, GIS ≥ 42) to improve clinical decision-making and treatment stratification, validating biomarkers like SBS39, miR-622, and metabolomic profiles in large-scale trials (n > 1000)
  ○ Develop scalable, cost-effective assays like GIScar, OGM, and aCGH to enhance sensitivity (70.8–77%) and reduce costs
  ○ Investigate methylation-based HRD markers and resistance mechanisms (e.g., *BRCA* reversion mutations, *SETD1A/EME1*, *SOX5*) to expand applicability
  ○ Integrate liquid biopsy approaches (e.g., cfDNA-based DirectHRD, shallow WGS) and multi-omics (transcriptomic signatures) to enhance accessibility and monitor dynamic HRD status
  ○ Investigate combination therapies (e.g., PARPi with ATR inhibitors, pembrolizumab) to overcome resistance mechanisms
  ○ Extend HRD testing to non-traditional cancers (e.g., gastrointestinal, colorectal, NSCLC) and early-stage tumors to broaden PARPi indications
  ○ Address logistical barriers through assay reimbursement, oncologist education, and cross-institutional standardization to ensure broader adoption

This roadmap integrates genomic, functional, and epigenetic assays with AMP-guided standardization to ensure accurate HRD detection and equitable therapeutic outcomes, advancing precision oncology across diverse populations.

## 14. Future Challenges

HRD-targeted therapies, particularly PARPi, face significant challenges in resistance mechanisms, standardization, broader application, biomarker development, and logistical barriers, limiting their optimal use in precision oncology. Addressing these challenges through prioritized research is critical to enhancing HRD’s therapeutic potential, as outlined in Section “Methodology” and Section 12, and Table 11.

### 14.1. Resistance Mechanisms

PARPi resistance undermines long-term efficacy, particularly in HGSOC and TNBC. *BRCA1/BRCA2* reversion mutations, detected in 20–40% of resistant HGSOC cases via circulating tumor DNA (ctDNA), restore HR function, negating PARPi sensitivity. Drug efflux pump upregulation (e.g., *MDR1*) and alternative repair pathways, such as NHEJ, contribute to resistance in 20–30% of HGSOC cases. In TNBC, replication fork protection via *53BP1* or *REV7* loss reduces PARPi efficacy. Other mechanisms, including *RAD51* overexpression and Wnt/β-catenin activation, are implicated in colorectal and non-small-cell lung cancer (NSCLC). *ZNF251* haploinsufficiency restores HR via *RAD51* upregulation, further complicating PARPi response. *SETD1A/EME1* downregulation and *SOX5* overexpression are novel resistance biomarkers, necessitating combination therapies with ATR or immune checkpoint inhibitors Conversely, PLK1 overexpression enhances PARPi sensitivity by suppressing HR, offering a potential strategy to counter resistance Murai and Pommier (2023) emphasized dynamic HRD assessment to monitor reversion mutations and *MDR1* upregulation. Laboratories should routinely assess reversion mutations in recurrent tumors post-PARPi/platinum therapy to guide treatment adjustments, with combination strategies (e.g., PARPi with ATR inhibitors) showing preclinical promise but requiring clinical validation

### 14.2. Standardization Issues

Inconsistent HRD definitions and assay thresholds hinder uniform clinical application. Recent AMP consensus recommendations highlight the need for standardized gene panels (*BRCA1*, *BRCA2*, *PALB2*, *RAD51C*, *RAD51D*), scar definitions, and validation protocols to ensure consistent HRD detection. Myriad myChoice CDx uses a quantitative HRD score (GIS ≥ 42), while FoundationOne CDx relies on qualitative somatic *BRCA* status/LOH, complicating cross-assay comparisons. Caris HRD’s WES-based GSS (≥42) achieves > 97% concordance with Myriad, supporting interchangeability but requiring harmonized thresholds. In breast cancer, subtype-specific GSS thresholds (e.g., 65% HRD in TNBC vs. 15% in luminal A) could harmonize detection but lack consensus. In ovarian cancer, *BRCA1*/*BRCA2* HRD detection is concordant across 20 assays, but non-*BRCA* HRD varies (correlation 0.4–0.9), risking 20% missed cases. Machine learning-based mutational signatures and multi-scale genomic features, such as HRProfiler (AUC > 0.90), reduce variability. Genome-wide LOH assays (55% TNBC HRD-positive) and low-pass WGS assays (DirectHRD, GSscan, shallowHRD, AcornHRD, AUC 0.980–0.997) offer reproducible, cost-effective alternatives. Oncomine’s 95.8% accuracy in HGSOC supports decentralized testing to minimize variability. *BRCA1* methylation enhances non-*BRCA* HRD detection, but assay variability persists. OGM and high-resolution aCGH capture 70.8% and 77% of HRD signatures, respectively, but require standardized thresholds. Marconato et al. (2025) advocated integrating RAD51 foci with shallow WGS to standardize non-*BRCA* HRD detection. Population-specific signatures in Asian patients and epigenetic alterations in male breast cancer further complicate standardization. Doig et al. (2023) emphasized pathologists’ role in standardizing interpretation protocols [Standardization efforts should prioritize cross-assay validation, subtype-specific thresholds, and detailed clinical reporting (e.g., assay limitations, HRR genes, biallelic status, methylation results) to ensure equitable PARPi access].

### 14.3. Broader Application

Extending HRD therapies beyond ovarian and breast cancers remains underexplored. HRD prevalence in colorectal (10–15%), pancreatic (15–20%), NSCLC (5–10%), and prostate (20–25%) cancers suggests therapeutic potential, with trials like TRITON2 showing olaparib PFS of 8.1 months in *BRCA2*-mutated prostate cancer. *BRCA2* mutations occur in 5–10% of prostate cases, and *RAD51C/RAD51D*, *BRIP1*, or *BARD1* defects may broaden eligibility across tumor types [65,66]. In breast cancer, HRD in luminal and HR+/HER2- subtypes, and *BRCA2/RAD51C* hypermethylation in male breast cancer (~30% prevalence), warrant further trials. Lower HRD rates in endometrial cancers (10–15%) and assay insensitivity to non-*BRCA* genes (e.g., *ATM*, *CDK12*) demand cancer-specific validation. Ren et al. (2025) confirmed biallelic *BRCA1/BRCA2*, *RAD51C*, *RAD51D*, *PPP2R2A*, and *TP53* alterations predict PARPi response in Asian cohorts [5]. Fiegl et al. (2024) highlighted *BRCA1* methylation as a universal HRD marker [6]. Jeon et al. (2025) identified 16% of non-*BRCA* breast cancers as HRD-positive, expanding PARPi candidates. Herzog et al. (2023) emphasized HRD’s potential in pancreatic and prostate cancers. Jiang et al. (2025) showed HRD’s prognostic and predictive value in gastrointestinal cancers, broadening PARPi indications for *BRCA*-wild-type cases. Clinical trials should prioritize non-*BRCA* HRD cancers, leveraging multi-omics profiling and methylation analysis to identify eligible patients.

### 14.4. Biomarker Development

Current HRD tests miss monoallelic or non-*BRCA* HRD cases, with non-*BRCA* HRD detection varying (60–70% concordance across 20 assays) in ovarian cancer. Next-generation tools like HRDetect, RAD51 foci assays, and HRProfiler (AUC > 0.90) improve sensitivity for *RAD51C/RAD51D*, *BRIP1/BARD1*, or *ATM/CHEK2* defects. Subtype-specific GIS/GSS thresholds and CNV integration in Chinese patients enhance precision. *BRCA1* methylation predicts 99% platinum sensitivity, and m6A/m5C dysregulation (*METTL3*, *ALKBH5*, *IGF2BP2*) promotes PARPi sensitivity via R-loop accumulation. GIScar, integrating RAD51 foci and serum miR-622, achieves 85% accuracy for HRD detection in HGSOC. Low-pass WGS assays (AUC 0.980–0.997) and the NanoString-based HRD200 classifier offer cost-effective solutions [36,37,38,39]. OGM and aCGH capture 70.8% and 77% of HRD cases, respectively. The Archer™ VARIANTPlex HRD and OncoScan’s SNP arrays integrate DNA/RNA methylation for robust detection but require validation. Emerging biomarkers, such as SBS39, miR-622, and metabolomic profiles, and AI-driven tools like DirectHRD, require large-scale validation (n > 1000) to improve non-*BRCA* HRD detection. Biomarker development should integrate functional (e.g., RAD51 foci), genomic (e.g., SBS39), and epigenetic (e.g., *BRCA1/RAD51C* methylation) markers, with validation in diverse cohorts.

### 14.5. Logistical Challenges

High costs (e.g., WGS USD 5000–USD 10,000), limited tissue availability, and integration of complex genomic testing into routine care impede HRD therapy delivery, particularly in low-resource settings. AI-based histologic analysis and cost-effective diagnostics like low-pass WGS and HRProfiler address these barriers. Higher HRD prevalence in Black and Asian populations necessitates equitable testing. Marconato et al. (2025) advocated decentralized testing to improve access, with Oncomine’s 95.8% accuracy supporting in-house solutions. The Archer™ VARIANTPlex HRD and OncoScan’s SNP arrays require cost reduction for broader adoption. Inconclusive test results (5–10% of cases) complicate decision-making, requiring repeat testing or alternative therapies. Genetic counseling and patient education address ethical implications, ensuring informed decision-making. Detailed clinical reports, including assay limitations, HRR genes, biallelic status, and methylation results, enhance transparency and provider decision-making. Liquid biopsy and shallow WGS approaches, with 90% sensitivity at 1% tumor fraction, and multi-omics integration enhance accessibility and scalability.

Key Points—Section 14: Future Challenges

- Resistance from *BRCA* reversion mutations and *SOX5* overexpression requires combination therapies.
- AMP-guided standardization of gene panels and scar definitions reduces assay variability.
- HRD’s therapeutic potential extends to gastrointestinal cancers, with validation needed.
- Liquid biopsy, shallow WGS, and multi-omics enhance accessibility and dynamic HRD detection.

## Figures and Tables

**Table 1:** A summary of the key differences between the double-strand break repair (DSBR) and synthesis-dependent strand annealing (SDSA) pathways in homologous recombination.

Feature	DSBR (Double-Strand Break Repair)	SDSA (Synthesis-Dependent Strand Annealing)
Holliday Junctions	Involves the formation of Holliday junctions, cross-stranded intermediates.	Does not involve Holliday junctions; no crossover intermediates.
Crossover vs. Non-Crossover	Can result in both CO and NCO products, depending on resolvase cleavage orientation.	Primarily results in NCO products, avoiding crossovers.
Genetic Diversity	Increases genetic diversity through CO events, exchanging genetic material,	Maintains genetic stability by avoiding CO, preserving parental genes.
Cellular Context	Crucial during meiosis for genetic diversity in gametes; less favored in somatic cells.	Preferred in somatic cells for accurate repair; less relevant in meiosis.
Mechanism	Strand invasion forms Holliday junctions: resolvases cleave junctions symmetrically (CO) or same orientation (NCO).	Strand invasion, DNA synthesis; newly synthesized strand displaces and anneals
Outcome Determination	Depends on the cleavage pattern of resolvases.	Inherently NCO due to displacement and annealing.
Risk of Genomic Alteration	Higher risk of LOH or rearrangements due to CO events.	Lower risk; promotes fidelity to the original sequence.
Cell Cycle Relevance	Active in S and G2 phases, prominent in meiotic prophase I.	Active in S and G2 phases of somatic cells, prioritizing stability.
Biological Role	Ensures chromosome segregation and diversity in gametes	Ensures high-fidelity DSBs repair in mitotic cells.
Key Proteins Involved	Involves resolvases (e.g., *GEN1*, *MUS81-EME1*) for junction resolution, plus RAD51 for strand invasion.	RAD51 for strand invasion, helicases for displacement.

**Table 2:** Overview of DNA repair mechanisms: functions, damage types, and characteristics.

Repair Mechanism	Primary Function	Type of Damage Repaired	Key Proteins/Pathways	Cell Cycle Phase	Fidelity
Homologous Recombination Repair (HRR) [30]	Repairs DSBs with high accuracy using homologous template.	Double-strand breaks (DSBs), interstrand crosslinks	BRCA1, BRCA2, RAD51	S and G2	High
Base Excision Repair (BER) [31]	Removes damaged bases, repairs SSBs.	Oxidized, alkylated, or deaminated bases	Glycosylases, APE1, DNA polymerase β	Throughout	High
Nucleotide Excision Repair (NER) [32] -Global genome NER -Transcription-coupled NER	Removes bulky DNA lesions.	UV-induced lesions, chemical adducts	XPA, XPC, ERCC1 (GG-NER, TC-NER)	Throughout	High
Mismatch Repair (MMR) [25]	Corrects replication errors.	Mismatched bases, insertion/deletion loops	MSH2, MLH1, PMS2	Post-replication (S)	High
Nonhomologous End-Joining (NHEJ) [22,23,25]	Ligates broken DNA ends.	Double-strand breaks (DSBs)	Ku70/80, DNA-PKcs, Ligase IV	G1	Error-prone
Translesion Synthesis (TLS) [33]	Bypasses DNA lesions during replication.	Unrepaired lesions (e.g., UV damage, adducts)	Specialized polymerases (Pol η, Pol ζ)	S	Low
Interstrand Crosslink (ICL) Repair [29]	Repairs covalent DNA strand links.	Interstrand crosslinks	Fanconi anemia (FA) pathway (FANCD2, FANCI)	S and G2	High

Notes: Summarizes DNA damage response (DDR) pathways, highlighting HRR’s high fidelity compared to error-prone NHEJ, which predominates in HRD. **Cell Cycle Phase:** Some mechanisms (e.g., HRR, NHEJ) are phase-specific due to template availability or cellular priorities, while others (e.g., BER, NER) operate throughout the cycle. Fidelity: High-fidelity pathways (HRR, BER, NER, MMR, ICL repair) minimize errors, while NHEJ and TLS are more error-prone due to their mechanisms of action. Key Proteins: The proteins listed are representative examples; each pathway involves a broader complex of factors.

**Table 3:** Key biomarkers and genomic signatures of HRD phenotype.

Key Biomarkers	Description		
*BRCA1*/*BRCA2* Mutations	Germline or somatic mutations in *BRCA1* or *BRCA2*; most recognized causes of HRD [30]		
Genomic Scars	DNA damage patterns from HRD, including loss of heterozygosity (LOH), telomeric allelic imbalance (TAI), and large-scale state transitions (LST) [41,42,43]		
Mutational Signatures	Distinct mutation patterns (e.g., COSMIC Signature 3) identified via whole-genome sequencing, associated with HRD [44,45].		
Other HRR Gene Alterations	Mutations/deficiencies in non-*BRCA* HRR genes (e.g., *RAD51C*/*RAD51D*, *BRIP1*, *PALB2*, *PPP2R2A*, *TP53*, *PLK1*, *SETD1A*, *EME1*, *SOX5*) contributing to HRD [5,12,18,19,25,34,40].		
Epigenetic Modifications	Promoter hypermethylation (e.g., *BRCA1*, *RAD51C*) and RNA methylation (e.g., m6A via *METTL3*, *ALKBH5*, *IGF2BP2*) contributing to HRD [7,8,29,46,47].		
Functional Biomarkers	Serum miR-622 and metabolomic profiles predicting platinum/PARPi response [12].		
**Genomic Signatures of the HRD Phenotype**			
**Genomic Scars**	**Definition**	**Key Characteristics**	**HRD Thresholds/Criteria**
Loss of Heterozygosity (LOH)	Irreversible loss of one parental allele at a chromosomal locus, leading to absence of tumor suppressor genes.	Copy-loss LOH; Copy-neutral LOH; LOH size > 15 Mb (but < whole chromosome) correlates with HR gene deficiency. Chromosome-specific LOH (e.g., 8p, 17p) enhances detection [5].	- gLOH ≥ 14% = HRD (LOH_high_) [42,54,55,56]
Telomeric Allelic Imbalance (TAI)	Number of subtelomeric regions with allelic imbalance (copy loss/gain) without crossing the centromere.	Linked to *BRCA* loss and cisplatin sensitivity and the result of stalled replication forks, increased replication stress; Enriched with 25 Kb CNVs, non-random breakpoints.	- N_tAI_ ≥ 22 indicates cisplatin sensitivity in wild-type BRCA tumors [43]
Large-Scale State Transitions (LST)	Chromosomal breaks between adjacent regions > 10 Mb (e.g., deletions, inversions, translocations).	Mostly translocations with high GC-content; detectable via OGM and aCGH [41,42].	- ≥15 LSTs (near-diploid) or ≥20 LSTs (near-tetraploid) = HRD (LST-high) [41]

**Table 9:** Myriad myChoice CDx and Caris HRD applications and findings.

Study	Cancer Type/Subtype	HRD Prevalence/Threshold	Key Findings
Quesada et al. (2025) [60]	Ovarian (HGSOC)	50–51% (≥42)	Global consensus, compares CDx assays, advocates standardization
Li et al. (2025) [80]	Ovarian/Breast	Adjusted thresholds	*ZNF251* haploinsufficiency may cause false negatives, suggests additional markers
Barnicle et al. (2024) [62]	Ovarian (HGSOC)	48–53% (≥42)	Consistent across 6 olaparib trials, reinforces PARPi efficacy prediction
Torres-Esquius et al. (2024) [84]	Ovarian (*RAD51*C/D-mutated)	70–80% (≥42)	Detects non-*BRCA* HRD effectively
Min Wang et al. (2023) [61]	Ovarian (Chinese HGSOC)	52% (≥38)	CNVs improve sensitivity, 97% platinum sensitivity in HRD+ *BRCA*m
Christinat et al. (2023) [68]	Ovarian (HGSOC)	49–53%	Normalized LST correlates with olaparib response, streamlined alternative
Capoluongo et al. (2022) [67]	Ovarian (HGSOC)	55–60%	Genomic + functional assays improve sensitivity over genomic-only
Fumagalli et al. (2022) [66]	Ovarian (HGSOC)	~50% (≥42)	High concordance with AmoyDx HRD Focus panel, in-house feasibility
Quesada et al. (2022) [59]	Ovarian (HGSOC)	50–51% (≥42)	Reliable for *BRCA1/2* and scars, limited for non-*BRCA* (e.g., *RAD51C*)
Weichert et al. (2022) [64]	Ovarian (HGSOC)	49–52% (≥42)	92% PPA (*BRCA1/2*), 87% (HRD score) with NGS kit harmonization
Wu et al. (2020) [65]	Ovarian (HGSOC)	51%	HRD score (89% sensitivity, 85% specificity) as robust alternative
Jiao et al. (2019) [112]	Ovarian (HGSOC)	52%	ASGAD algorithm achieves 93% PARPi response accuracy
Caris Life Sciences (2022) [113,114]	Ovarian (HGSOC)	50–53% (GSS ≥42)	WES-based GSS (LOH + LST) achieves >97% concordance with Myriad myChoice CDx
Engebrethsen et al. (2023) [76]	Breast (Luminal)	15–25% (≥42)	Links high scores to replication stress and *BRCA1/2* defects
Yndestad et al. (2023) [75]	Breast (HR+/HER2-, HER2+)	15–20% (HR+/HER2-), 30–35% (HER2+)	Validates utility across diverse subtypes
Feng et al. (2023) [81]	Breast	Correlates with GSS	Genomic scar score (GSS) aligns with LOH, TAI, LST, enhancing precision
Lenz et al. (2023) [69]	Breast (TNBC, HER2+, Luminal)	65% (TNBC), 40% (HER2+), 25% (Lum B), 15% (Lum A)	GIS complements HRD score, reflecting subtype-specific instability
Jacobson et al. (2023) [70]	Breast	~45%, TNBC 70% (≥42)	Multi-scale features (e.g., tandem duplications) enhance subtle HRD detection
Lim et al. (2023) [72]	Breast	~50%, TNBC 60–70%	Machine learning mutational signatures distinguish *BRCA1/2*-driven HRD
Batalini et al. (2023) [53]	Breast	High scores	Captures somatic *BRCA1/2* and germline *PALB2* HRD, aligns with PARPi response
Zhang et al. (2022) [71]	Breast (Early TNBC)	50–60% (≥42)	Predicts pCR with platinum neoadjuvant therapy
André et al. (2020) [78]	Male Breast Cancer	~30%	Suggests adaptation with epigenetic markers (*BRCA2/RAD51C* hypermethylation)

Notes: Summarizes applications of Myriad myChoice CDx and Caris HRD, with thresholds (GIS ≥ 42, GSS ≥ 42) and findings. Caris HRD’s WES-based approach enhances detection in non-*BRCA* cases and extends to breast, prostate, and pancreatic cancers [107].

**Table 10:** FoundationOne CDx applications and findings.

Study	Cancer Type	HRD Detection	Key Findings
Weichert et al. (2022) [64]	Ovarian (HGSOC)	49–52% (*BRCA1/2*, LOH)	Effective for *BRCA1/2* and LOH, limited for non-*BRCA* (e.g., *RAD51C*) [60]
Weichert et al. (2022) [64]	Breast (TNBC)	50–70% (*BRCA1*-driven)	Captures *BRCA1* HRD, requires optimization for non-*BRCA* genes [60]
Chien-Feng Li et al. (2022) [81]	Breast (TNBC)	55% (LOH-based)	Genome-wide LOH assay aligns with F1CDx, offers cost-effective alternative [73]
Quesada et al. (2025) [60]	Ovarian (HGSOC)	50–51% (*BRCA1/2*, LOH)	Compares with myChoice CDx, supports standardization for PARPi eligibility [64]
Marconato et al. (2025) [15]	Ovarian/Breast	Varies by assay	Broader genomic profiling, limited non-*BRCA* HRD specificity compared to dedicated assays [89]

**Table 11:** Components of the conceptual model for HRD detection and treatment.

Component	Description	Key Features
Genomic and Functional Assay Integration	Combines genomic (NGS, SNP arrays) and functional (RAD51 foci, DNA fiber assay) assays for comprehensive HRD detection	Captures *BRCA1/BRCA2* and non-BRCA HRD; addresses dynamic HRD status with GIScar, Oncomine, OGM, aCGH
Multi-Omics Profiling	Uses machine learning to integrate genomic, transcriptomic, proteomic data for precise HRD subtyping	Enhances sensitivity (e.g., HRDetect > 90%, ASGAD 93%) with HRProfiler, DirectHRD, GSscan, shallowHRD, AcornHRD
Methylation Analysis	Assesses *BRCA1/RAD51C* promoter hypermethylation in mutation-negative cases; Epitranscriptomics (RNA) modifications/methylation	Identifies HRD in 1–15% ovarian, up to 60% TNBC cases; includes m6A/m5C dysregulation and methylations driven DDR genes
Biallelic Inactivation Reporting	Reports biallelic status of HRR gene mutations to predict PARPi response	Enhances outcome prediction (>80% biallelic rate in *BRCA*-associated tumors)
Resistance Mitigation	Targets resistance mechanisms (e.g., *BRCA* reversion, *ZNF251* haploinsufficiency) via combination therapies	PARPi with ATR or immune checkpoint inhibitors; addresses *SETD1A/EME1*, *SOX5* resistance
Standardization	Harmonizes assay thresholds (e.g., GIS ≥ 42, gLOH ≥ 16%) and reporting protocols	Reduces non-*BRCA* HRD variability (60–70% concordance); includes OGM, aCGH, low-pass WGS
Clinical Translation	Translates detection into practice with cost-effective diagnostics and genetic counseling	Ensures equitable access; addresses disparities in Asian, Black populations
Validation Roadmap	Validates model across diverse tumor types and populations	Prioritizes large-scale trials (n > 1000) for novel biomarkers (e.g., SBS39, miR-622, metabolomic profiles)

Notes: The model integrates multi-omics, methylation, and biallelic reporting to advance precision oncology, emphasizing novel tools (e.g., Archer™, OncoScan, low-pass WGS) and biomarkers to enhance detection and therapeutic outcomes.
